# Prospects and challenges in developing the next generation astronomical sub-millimetre and terahertz heterodyne receivers

**DOI:** 10.1007/s10509-026-04605-0

**Published:** 2026-07-29

**Authors:** Boon-Kok Tan

**Affiliations:** Department of Physics (Astrophysics), University of Oxford, Denys Wilkinson Building, Keble Road, Oxford, OX1 3RH Oxfordshire UK

**Keywords:** Superconductor–insulator–superconductor (SIS) mixers, Heterodyne receivers, Focal plane array, Simultaneous observing

## Abstract

The development of next-generation astronomical receivers operating at millimetre (mm), sub-mm, and terahertz frequencies is essential to meet the increasing demand for wide-field, high-spectral-resolution observations. In this work, we examine the prospects and challenges associated with advancing heterodyne receiver technologies, with particular emphasis on superconductor–insulator–superconductor (SIS) mixers. We present a series of technological pathways aimed at enhancing receiver performance, including ultra-broadband RF and IF SIS mixer designs, compact sideband-separating (2SB) architectures enabled by planar superconducting circuit integration, and scalable focal plane array (FPA) concepts capable of supporting hundreds to thousands of pixels. These developments are motivated by the need to significantly improve mapping speed and survey efficiency for future facilities such as ALMA upgrades, AtLAST, LST, and next-generation space missions. Another key contribution of this work is the introduction of simultaneous observing multi-band receivers (SOMBRs), which enable concurrent multi-band 2SB observations through minimal additional hardware by reconfiguring conventional receiver architectures. This approach allows continuous spectral coverage while preserving phase information required for interferometric applications. We discuss the principal challenges in realising these systems, including bandwidth optimisation, impedance matching, local oscillator distribution, and scaling to large-format arrays. Overall, this work outlines a viable pathway towards highly integrated, ultra-broadband, and scalable heterodyne receiver systems, which are expected to play a critical role in enabling the next generation of astronomical discoveries.

## Introduction

The field of far-infrared (FIR) astronomy and astrophysics, encompassing the millimetre (mm) to sub-millimetre (sub-mm) wavelength regime, has advanced substantially over the past few decades, driven by a series of transformative observational facilities. These include space-based missions such as the Infrared Astronomical Satellite (IRAS Neugebauer et al. 1984), the Spitzer Space Telescope (Werner et al. 2004), and the Herschel Space Telescope (Pilbratt et al. 2010), alongside numerous ground-based observatories, including the Atacama Large Millimetre/sub-millimetre Array (ALMA Wootten and Thompson 2009), the James Clerk Maxwell Telescope (JCMT Robson et al. 2017), the Large Millimetre Telescope (LMT Hughes et al. 2020), the Institut de Radioastronomie Millimétrique (IRAM) 30-m telescope (Baars et al. 1987), the Northern Extended Millimetre Array (NOEMA Chenu et al. 2016), and the Submillimetre Array (SMA Ho et al. 2004), among others. The suite of bolometric and spectroscopic (heterodyne) instruments deployed across these platforms has significantly expanded and accelerated our understanding of the FIR Universe relative to earlier decades.

For high-spectral-resolution FIR spectroscopic observations in the frequency range of approximately 0.1–1 THz, superconductor–insulator–superconductor (SIS) heterodyne mixers have emerged as the detectors of choice. These devices provide ultra-sensitive, coherent detection, with sensitivities approaching the quantum noise limit and exceptionally low added noise across wide radio-frequency (RF) and intermediate-frequency (IF) bandwidths (Blundell and Winkler 1991; Belitsky et al. 2022). Consequently, SIS mixer technology is employed in eight of the ten observing bands of ALMA, one of the most advanced mm/sub-mm observatories currently in operation (Tarenghi 2008).

However, following the completion of the first phase of ALMA and the Herschel Space Telescope, progress in SIS mixer development has slowed, largely owing to the absence of new large-scale international mm/sub-mm initiatives. This has contributed to a perceived stagnation within the community responsible for the design and fabrication of the quantum sensors underpinning many of these astronomical advances. In parallel, engagement from early-career researchers, including postgraduate students and postdoctoral research associates, has declined. Nevertheless, we argue that the present period represents a significant opportunity to reinvigorate and further advance superconducting heterodyne detector technologies for mm/sub-mm and THz astronomy.

Several recent and forthcoming developments highlight this renewed momentum. First, the ALMA-2030 Wideband Sensitivity Upgrade (WSU) programme aims to expand the IF (intermediate frequency, and where feasible, radio frequency (RF)) bandwidths of SIS mixers, a technically demanding yet potentially transformative objective. This effort is engaging both experienced contributors from the original ALMA project and a new generation of researchers, and is expected to deliver order-of-magnitude improvements in observational capability (Carpenter et al. 2022). Second, compact, large-format focal plane SIS mixer arrays are being considered for a prospective ALMA-2040 upgrade. Third, next-generation large-aperture sub-mm facilities, such as the Atacama Large Aperture Submillimeter Telescope (AtLAST Mroczkowski et al. 2025) and the Large Submillimeter Telescope (LST Ramasawmy et al. 2022), are anticipated in the coming decades, placing stringent demands on receiver technologies that exceed current performance by substantial margins. Fourth, since the decommissioning of Herschel, no FIR space missions have been operational. In response, the community has proposed several next-generation space observatories including the Space Infrared Telescope for Cosmology and Astrophysics (SPICA Swinyard and Nakagawa 2009), the Far Infrared Space Terahertz Telescope (FIRSTT Wiedner et al. 2026), and the Light Emission Terahertz Observatory (LETO Rigoupoulo et al. 2026); through major agencies such as NASA (National Aeronautics and Space Administration), ESA (European Space Agency), and JAXA (Japan Aerospace Exploration Agency). These missions will require compact, ultra-broadband mixer arrays with high sensitivity and stability. In addition, the Event Horizon Telescope (EHT) collaboration has demonstrated the scientific potential of coordinated mm/sub-mm observations, motivating the development of heterodyne receivers capable of simultaneous wideband operation for broadband spectral surveys and time-domain studies. Finally, prospective future concepts, such as space–space or ground–space FIR VLBI (very long baseline interferometry) (Johnson et al. 2024; Villard et al. 2026), will critically depend on the maturity of such heterodyne detection schemes.

Accordingly, this manuscript explores the opportunities and challenges associated with advancing superconducting heterodyne technology across several interrelated areas, in particular:

1. the development of broadband RF and IF SIS mixers to meet and exceed ALMA WSU requirements;
2. the implementation of novel technologies enabling large-format focal plane arrays (FPAs); and
3. the realisation of multi-band, simultaneous-observation receiver architectures.

Each of these advanced functionalities necessitates careful consideration across multiple aspects of SIS mixer chip design and development. Furthermore, the realisation of high-performance heterodyne receivers requires attention to a broader set of system-level components beyond the mixer chip itself. These include, for example, ultra-broadband feed horns and local oscillators (LOs) on the RF side, innovative LO injection schemes for compact large-scale arrays, and the bandwidth capabilities of IF readout chains, encompassing microwave interconnects and backend electronics. Achieving seamless system performance requires coordinated optimisation of all such components to match the capabilities of the SIS mixer. In this manuscript, however, we focus primarily on the mixer chip itself: the central element of the receiver system. Consideration of the associated ancillary technologies lies beyond the present scope, although we emphasise that their development will be essential and will benefit from sustained community-wide collaboration.

The central premise of this work is that, given the range of forthcoming facilities, including AtLAST, LST, ALMA upgrades, JCMT developments, EHT extensions, and prospective FIR space missions such as LETO, SHARP, and FIRSST, there is a timely opportunity to revitalise international efforts in mm/sub-mm heterodyne receiver development.

## Ultra-broad RF & IF bandwidth double-sideband SIS mixers

Since their inception and first experimental demonstration, SIS mixers have been deployed in nearly all mm/sub-mm observatories, enabling a wide range of spectroscopic observations that were previously inaccessible. These devices have facilitated numerous significant scientific discoveries, including, most notably, the direct imaging of a black hole event horizon (Collaboration EHT et al. 2019). It has become customary to define the accessible mm/sub-mm atmospheric transmission windows, spanning approximately 30 GHz to 1 THz, based on the atmospheric conditions of the Atacama Desert, the site of ALMA.

To provide context for this manuscript, we consider ALMA as a reference system. The observatory is equipped with ten receiver bands housed within a common receiver cabin,^1^ collectively covering the full mm/sub-mm wavelength range. These receivers are largely based on mature SIS mixer technologies, with the exception of Bands 1 and 2, which employ high electron mobility transistor (HEMT) amplifiers as first-stage detectors. Each SIS-based receiver (Bands 4–10) is polarisation-sensitive, achieved by combining two single-polarisation receiver chains using an RF polariser. Bands 4–8 utilise sideband-separating (2SB) architectures, providing an intermediate-frequency (IF) bandwidth of 4 GHz per polarisation per sideband, except for Band 6, which offers 5.5 GHz bandwidth. The two highest-frequency bands (Bands 9 and 10) employ double-sideband (DSB) configurations, each with an IF bandwidth of 8 GHz per polarisation; these are anticipated to be upgraded to 2SB architectures in the near future. Each SIS receiver band covers approximately 26% fractional RF bandwidth, with the exception of Bands 9 and 10, which achieve approximately 18%. Overall, system sensitivities approach a few times the quantum noise limit across nearly the full operational RF range of ALMA.

ALMA, along with other contemporary mm/sub-mm facilities operating in high-R (spectral resolution) modes, has delivered substantial scientific output using these established SIS mixer technologies. Looking ahead, a major priority, driven initially by the ALMA community is to enhance receiver sensitivity and, more critically, to significantly expand the IF bandwidth. The ALMA Wideband Sensitivity Upgrade (WSU Carpenter et al. 2022) programme aims to increase IF bandwidth by at least a factor of two, with a factor of four considered highly desirable. In addition, there is strong motivation to extend RF bandwidth as far as practicable in next-generation SIS mixers. Improvements in both sensitivity and bandwidth will have broad implications for future mm/sub-mm spectroscopic instruments beyond ALMA.

In this section, we focus on the simplest class of SIS mixer architectures, namely single-ended double-sideband (DSB) designs, which can subsequently be extended to dual-polarisation 2SB receiver systems. We first outline recent efforts to advance the state of the art in DSB SIS mixer technology, before considering more complex architectures. The discussion is organised around three key development directions:

- Expansion of RF bandwidth, with the goal of combining adjacent ALMA frequency bands;
- Extension of IF bandwidth, without necessarily targeting ultra-wide RF coverage; and
- Simultaneous realisation of broad RF and IF bandwidth in DSB mixer designs.

For consistency, the majority of mixer designs discussed in the following sections are based on a common set of technological parameters, unless stated otherwise. These include:

- A silicon-on-insulator (SoI) substrate with a 10 μm device layer, incorporating gold beam leads for electrical interfacing;
- Standard Nb–AlO_*x*_–Nb (niobium-aluminium oxide-niobium) superconducting tunnel junctions with a critical current density of approximately 8–10 kA cm^2^; and
- A superconducting ground plane comprising 250 nm of Nb, and a wiring layer of 450 nm Nb, separated by a silicon monoxide (SiO) dielectric layer of approximately 400 nm thickness.

### Double-sideband single-ended SIS mixers

Since the successful deployment of SIS mixers in ALMA and the Herschel Space Telescope, there has been sustained motivation within the community to expand the RF bandwidth of these devices. Broadening the instantaneous observational bandwidth of individual receivers reduces the total number of receiver channels required to cover the full RF range of a telescope, which is particularly advantageous for space-borne FIR observatories, where constraints on mass, volume, and system complexity are stringent.

**Fig. 1:**
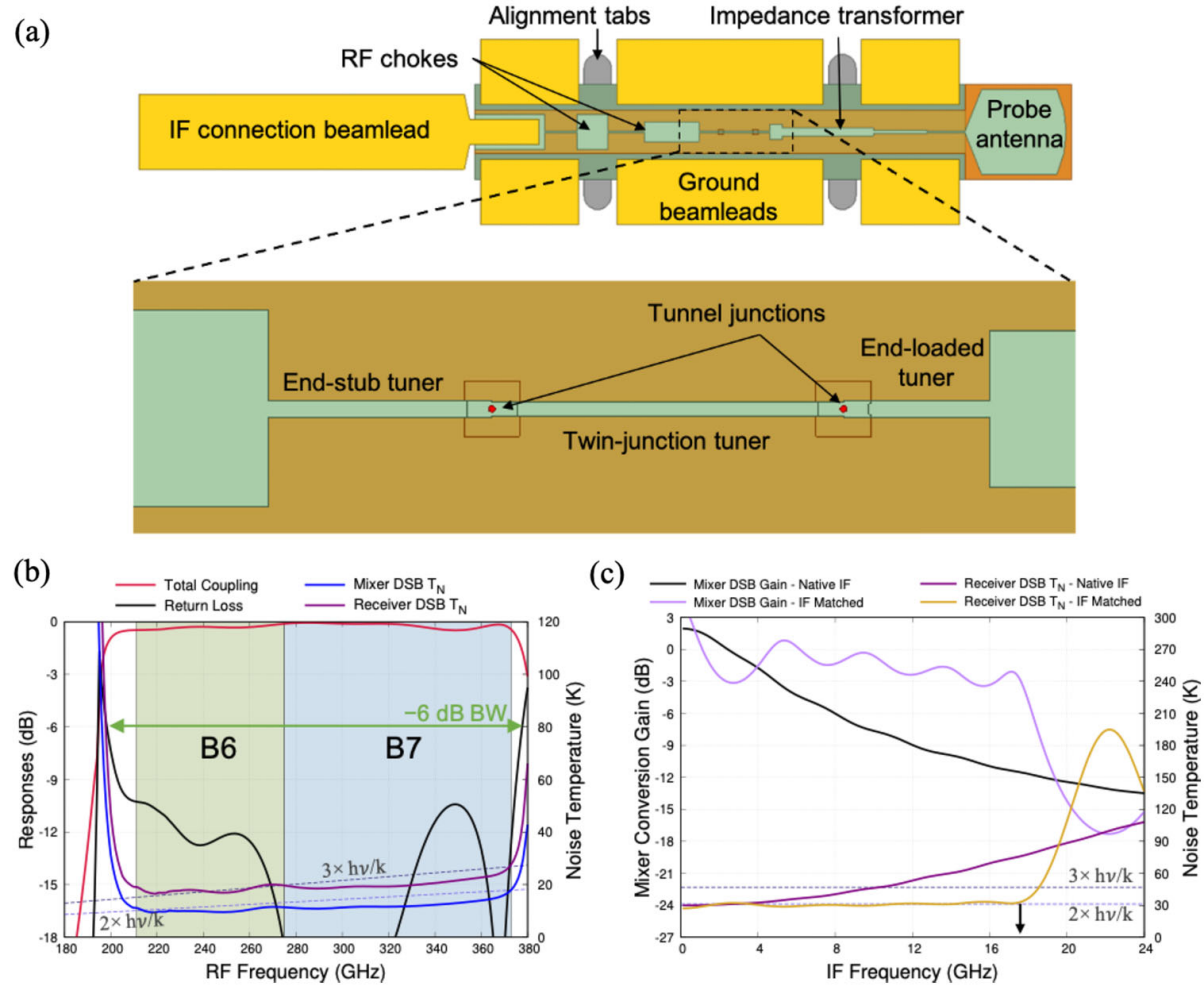
(a) Example of a broadband SIS mixer chip. (b) Simulated RF noise performance of the mixer. (c) Corresponding IF conversion gain and noise performance with and without an IF matching circuit (Kim et al. 2025; Tan et al. 2014, 2013a). The receiver DSB noise temperature in both plots is calculated assuming room-temperature (297 K) optics with 5% optical loss and a 3 K noise temperature first-stage cryogenic LNA

Figure 1 presents an example of a band-combining SIS mixer covering the RF ranges of ALMA Bands 6 and 7, employing a radial probe antenna. The design integrates end-loaded and end-stub tuning techniques (Kooi 2008) with a twin-junction configuration to achieve RF bandwidths extending beyond those of a single ALMA band. The electromagnetic performance of the mixer chip is simulated using Ansys High Frequency Structure Simulator (HFSS), while the heterodyne quantum mixing characteristics are evaluated using SuperMix (Ward et al. 1999; Rice et al. 1999), a numerical tool developed at the California Institute of Technology based on Tucker theory (Tucker and Feldman 1985). A similar simulation framework (Tan 2015) is employed for all mixer designs presented in this manuscript.

As shown in Fig. 1(b), the simulated total power coupling from the full-height input waveguide (385 μm × 770 μm) to the tunnel junctions approaches unity across the combined Band 6 and 7 frequency range. The corresponding predicted intrinsic double-sideband (DSB) noise temperature of the mixer chip remains below twice the quantum limit across this bandwidth. Here, we estimate the receiver noise temperature incorporating a realistic system assumptions, namely 5% optical losses at room temperature (297 K) and a cryogenic low-noise amplifier (LNA) with a noise temperature of 3 K, to take into account the impact of the mixer conversion gain. As indicated by the purple curve in Fig. 1(b), the receiver achieves noise performance close to three times the quantum limit while simultaneously covering both ALMA Bands 6 and 7.

**Fig. 2:**
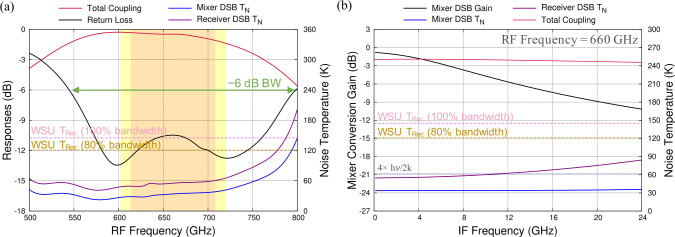
(a) Predicted RF performance of a wideband SIS mixer covering the ALMA-defined Band 9 frequency range, based on a topology similar to Fig. 1(a). (b) Corresponding IF performance, demonstrating bandwidth exceeding 20 GHz under ALMA-WSU requirements. The receiver DSB noise temperature () $T_{ \mathrm{N}}$) in both plots is calculated assuming room-temperature (297 K) optics with 5% optical loss and a 3 K noise temperature cryogenic LNA

The same design principles, combining end-loaded and end-stub impedance tuning with twin-junction configurations can, in principle, be extended to broader band-combination strategies across the full suite of ALMA frequency bands. Figure 2 illustrates a further example targeting the frequency range of ALMA Band 9, employing a similar radial probe antenna architecture. At higher frequencies, RF bandwidth expansion becomes increasingly challenging due to the elevated junction capacitance (i.e., increasing ωC). In this design, tunnel junctions with a diameter of 1 μm are employed in conjunction with a half-height rectangular waveguide to facilitate broader bandwidth operation. The increased aspect ratio of the waveguide reduces its characteristic impedance, thereby improving impedance matching between the waveguide and the junctions over an extended RF range.

Using the ALMA-WSU noise temperature requirements as a benchmark, the simulated performance shown in Fig. 2(a) indicates that the mixer can, in principle, satisfy these requirements over nearly twice the original ALMA Band 9 bandwidth, extending from 602–720 GHz to approximately 550–780 GHz.^2^

### IF bandwidth consideration

The mixer designs presented above primarily employ well-controlled on-chip microstrip transmission-line circuitry to achieve broad RF bandwidth performance. Similar approaches have been demonstrated previously by Kojima et al. (2017), who reported a 275–500 GHz wideband waveguide SIS mixer combining ALMA Bands 7 and 8. In that work, a fractional RF bandwidth of approximately 60% was achieved, with measured noise temperatures around three times the quantum limit, using both single- and twin-junction tuning schemes with nominal junction diameters of 0.8 and 0.9 μm. However, as noted in that study, the achievable IF bandwidth was comparatively limited. This limitation arises fundamentally from the nature of the on-chip microstrip circuitry: at IF frequencies, these structures behave predominantly as parallel-plate capacitors, thereby imposing an intrinsic constraint on the IF bandwidth.

To mitigate this limitation, IF impedance matching techniques may be employed to broaden and flatten the IF gain and noise response. Such matching networks can be implemented either off-chip (Tan et al. 2014, 2013a), using conventional printed circuit board (PCB) technology, or on-chip using superconducting circuitry (Kooi 2008; Kojima et al. 2018). An example of an off-chip IF matching approach is illustrated in Fig. 1(c). In the absence of matching, the intrinsic mixer conversion gain degrades rapidly across the IF band, primarily due to the large microstrip structures required for broadband RF operation. This degradation leads to a substantial increase in receiver noise temperature at higher IF frequencies. However, with a suitably designed IF matching network, it is possible to compensate for the parasitic IF capacitance of the mixer chip (arising from both the junction capacitance and the on-chip RF circuitry) and to match the dynamic output impedance of the SIS mixer to a 50 Ω environment. This results in a significantly flatter conversion gain profile (Tan et al. 2013b) and improved noise performance, extending the usable IF bandwidth from approximately 10 GHz at three times the quantum limit to around 17.5 GHz at approximately twice the quantum limit.

Here, we adopt a realistic receiver noise model that includes 5% optical losses at room temperature (297 K) and a cryogenic low-noise amplifier (LNA) with a noise temperature of 3 K. This approach enables assessment not only of the intrinsic mixer performance but also of the impact of conversion gain on overall receiver sensitivity.

On-chip IF matching techniques have also been demonstrated by Kojima et al. (2018), achieving IF bandwidths of up to approximately 8 GHz. Compared with on-chip implementations, off-chip matching offers greater flexibility, as the matching network can be optimised post-fabrication to account for device variations and fabrication tolerances. However, this approach increases the physical footprint of the receiver module, which is disadvantageous for large-format array applications.

At higher RF frequencies, the physical dimensions of on-chip microstrip structures are reduced due to shorter wavelengths, thereby mitigating their impact on IF performance. This effect is illustrated in Fig. 2(b), where an IF bandwidth extending to approximately 12 GHz near the centre of the RF band is achieved.^3^ If a noise threshold of four times the quantum limit is adopted, the IF bandwidth is significantly extended. When evaluated against the ALMA-WSU noise specifications, the design indicates potential for IF bandwidths exceeding 20 GHz, assuming ideal fabrication and implementation.

For higher-frequency mixers, where the footprint of the on-chip microstrip circuits are generally much smaller because of the shorter RF wavelength operation, the impact on the IF bandwidth performance is rather tolerable. This is clearly shown in Fig. 2 (b), where the IF bandwidth covering up to 12 GHz near the centre of the RF band,^4^ if we use a 4× quantum noise limit as a guideline. Should we use the ALMA-WSU noise requirement as guideline, the IF performance appears to have much larger margin for good performance beyond 20 GHz, should the design can be realised perfectly during fabrication.

**Fig. 3:**
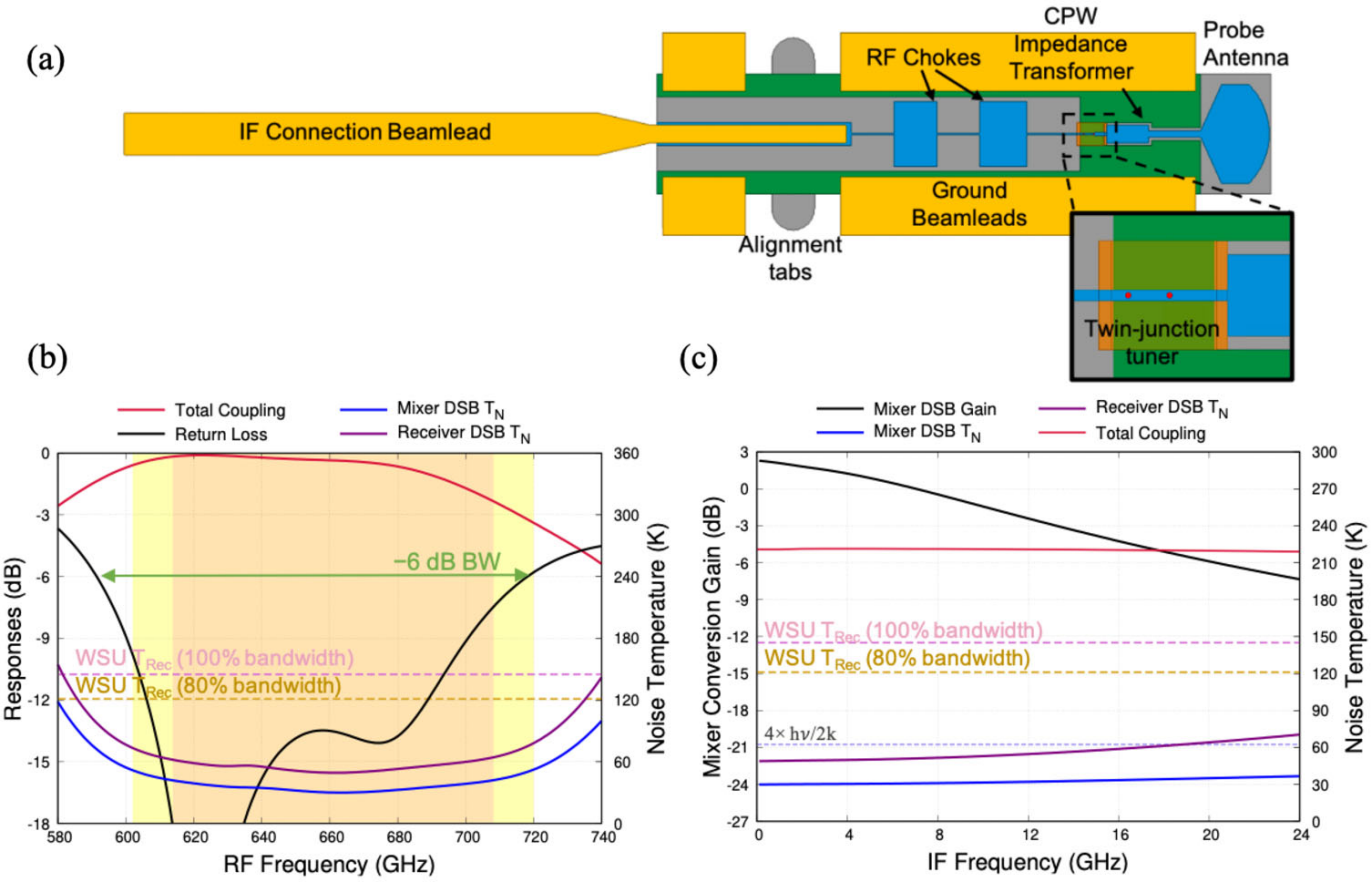
(a) Example of a CPW-based ALMA Band 9 SIS mixer chip. (b) Simulated RF noise performance of the mixer. (c) Corresponding intrinsic IF conversion gain and noise performance without an IF matching circuit. The receiver DSB noise temperature in both plots is calculated assuming room-temperature (297 K) optics with 5% optical loss and a 3 K noise temperature first-stage cryogenic LNA

An alternative approach to broadening the IF bandwidth involves modifying the transmission-line architecture of the mixer. Figure 3(a) presents an example in which the majority of on-chip microstrip lines are replaced with lower-capacitance coplanar waveguide (CPW) structures. This configuration substantially reduces parasitic capacitance, thereby enabling significantly wider IF bandwidths. However, the use of CPW transmission lines limits the ability to realise ultra-low impedance sections required for end-loaded and end-stub tuning techniques. Consequently, the flexibility to employ multiple tuning strategies for RF bandwidth expansion is reduced. This trade-off is evident in Fig. 3(b) and (c), where the IF bandwidth extends to approximately 19 GHz (assuming a 4× quantum noise limit), while the RF bandwidth is effectively constrained to the nominal ALMA Band 9 range when evaluated using a –6 dB return loss criterion.

### Four-probe dual-polarisation mixers

The examples presented thus far have focused on single-ended mixers operating in a single-polarisation configuration, representing the simplest form of SIS receiver architecture. Before considering more advanced operational modes, such as sideband separation, we first address the implementation of polarisation-sensitive receivers. While single-polarisation mixers offer simplicity, they are sensitive to only one linear polarisation component of the incident electromagnetic field. Consequently, approximately half of the available RF power is not detected. To maximise receiver sensitivity, and thereby reduce required observation time while enhancing scientific return, it is essential to detect both orthogonal polarisation components of the incoming electromagnetic wave. This also enables measurement of the polarisation state of the astronomical signal as an additional scientific observable.

Conventionally, dual-polarisation (i.e., full-sensitivity) heterodyne receivers are realised by combining two SIS mixers with an orthomode transducer (OMT), which separates the incident signal into two orthogonal linear polarisations prior to down-conversion. OMTs may be implemented using a variety of approaches, most commonly waveguide-based magic-T structures or quasi-optical polarisers. However, these solutions typically require substantial physical volume to accommodate waveguide junctions or free-space optical components.

A more compact architecture has been demonstrated by Shan et al. (2019) and Wenninger (2023), in which these bulky components are replaced by an on-chip superconducting four-probe antenna positioned along the axis of a circular waveguide. This configuration offers a significantly reduced footprint, making it particularly attractive for compact receiver designs and large-format array implementations. An additional advantage of this approach is the elimination of the need for a circular-to-rectangular waveguide transition, as the output of the feed horn is inherently circular.

As discussed in Sect. 2.2, certain applications do not require simultaneous optimisation of both RF and IF bandwidths. For example, in FIR space missions, SIS receivers may share backend IF spectrometers with supra-THz hot-electron bolometer (HEB) mixers, whose narrower IF bandwidth typically defines the overall system bandwidth. In such cases, the requirement for ultra-broad IF bandwidth in SIS receivers is relaxed. Similarly, for line-targeted observations where spectral features are known a priori, operation in DSB mode may be sufficient.

**Fig. 4:**
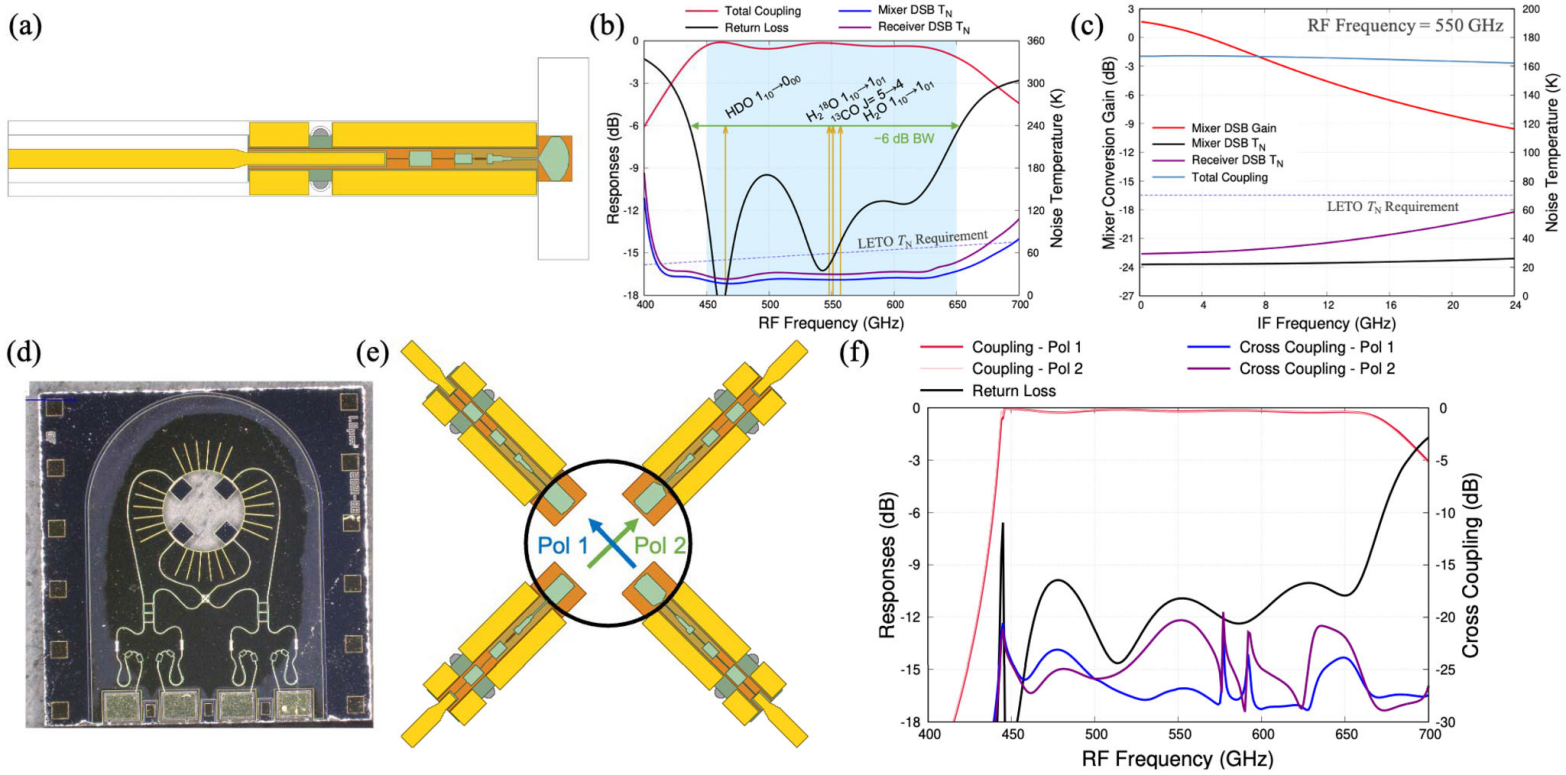
(a) Radial-probe SIS mixer chip optimised for the LETO Band 1 frequency range. Simulated (b) RF and (c) IF performance of the mixer. (d) Example of an on-chip dual-polarisation four-probe antenna mixer chip. (e) Layout of a four-chip dual-polarisation SIS receiver employing four identical mixer chips as shown in (a). (f) Predicted RF performance of the four-probe dual-polarisation receiver in (e). The receiver DSB $T_{\mathrm{N}}$ is calculated assuming 80 K optics with 5% optical loss and a 3 K noise temperature cryogenic LNA

In this context, we consider the example of the Band 1 SIS receiver proposed for LETO, currently under evaluation within the ESA M8 mission programme. This example illustrates the implementation of a dual-polarised DSB receiver architecture, which may also be extended to large-format arrays (see Sect. 3). Figure 4(a)–(c) present the simulated performance of a mixer design based on the radial-probe microstrip architecture described previously, optimised for the LETO Band 1 frequency range. The RF and IF performance matches the prediction of those of the single-ended devices.

Figure 4(d) shows a fully integrated dual-polarisation DSB SIS mixer realised on a single chip, as investigated by Wenninger (2023). While this approach demonstrates promising performance, it entails considerable design and fabrication complexity. In particular, the integration of multiple junctions (e.g., eight in the example shown) requires precise and simultaneous optimisation, placing stringent demands on fabrication tolerances. Furthermore, the large chip area required complicates the mechanical design of the mixer block, particularly with regard to minimising RF leakage through the gaps necessary to accommodate the chip.^5^ Although such issues may be mitigated through the use of ultra-thin SoI or silicon nitride (SiN) (Liu et al. 2015) membrane technologies, a simpler alternative is to employ multiple individual mixer chips within the same four-probe architecture. This approach, previously demonstrated by Liu et al. (2015), confines the required mechanical gaps to the immediate vicinity of each chip.

Figures 4(e) and (f) illustrate this concept, in which four identical mixer chips are fabricated on 10 μm thick SoI substrates and arranged within the four-probe configuration. The individual mixer layouts are largely based on the single-ended designs discussed earlier, with modifications to the probe antenna geometry and microstrip impedance transformer. The results demonstrate that transitioning from a single-ended rectangular-waveguide-fed design to the four-probe circular-waveguide architecture preserves the RF performance. Specifically, the total power coupling from the waveguide to each mixer element remains comparable to that of the single-ended configuration, with consistent performance across both polarisation channels. Cross-polarisation isolation is maintained below –20 dB.

These results indicate that radial-probe SIS mixer designs can be effectively extended to dual-polarisation, full-sensitivity operation with minimal additional optimisation. However, this architecture does require the inclusion of an IF power-combining network to combine the down-converted signals from the individual mixer elements within each polarisation channel.

### Prospects & challenges

All of the examples presented above employ two tunnel junctions in parallel, but it is important to note that the choice between single- and twin-junction configurations has direct implications for both RF and IF bandwidth performance. Single-junction mixers generally exhibit broader IF bandwidth, as the effective junction capacitance is approximately half that of a twin-junction configuration. Conversely, twin-junction mixers are less sensitive to fabrication tolerances and typically support wider RF bandwidths due to increased design flexibility in impedance matching. However, this advantage is offset at IF frequencies, where the increased capacitance degrades the achievable bandwidth.

These considerations indicate that there remains substantial scope for further optimisation of SIS mixer designs to simultaneously achieve ultra-broad RF and IF bandwidths. Such advances would not only reduce system complexity, hardware footprint, and associated logistical constraints for both space-borne and ground-based observatories, but also enable enhanced scientific capability through the simultaneous observation of multiple spectral features across an extended frequency range.

Several technological avenues may facilitate such improvements, including:

- the use of higher aspect ratio waveguides (e.g., beyond 2:1, as explored in Kojima et al. 2018) to further reduce the impedance mismatch between the input waveguide and the tunnel junctions;
- the development of high critical current density junctions, achieved through thinner AlO_*x*_ barriers or alternative materials such as AlN (aluminium nitride, Pavolotsky et al. 2026), to improve impedance matching towards a 50 Ω environment at IF frequencies;
- the implementation of sub-micron-scale junctions (Khudchenko et al. 2015; Tretyakov et al. 2024) to reduce junction capacitance and improve the ωRC product, thereby extending both RF and IF bandwidths; and
- the exploration of alternative junction architectures, such as series-connected SIS junctions (Tong et al. 2012).

For operation at frequencies beyond approximately 680 GHz, approaching or exceeding the superconducting gap frequency of niobium, additional considerations arise. At these frequencies, Nb-based transmission lines and junction electrodes exhibit increased loss, necessitating alternative materials and device concepts. Potential solutions include:

- the use of higher-gap superconductors to reduce RF losses at frequencies beyond the Nb gap; and
- the development of non-Nb-based tunnel junctions, such as niobium nitride (NbN) or niobium titanium nitride (NbTiN), or hybrid structures combining high-gap superconducting electrodes with Nb ground planes (Khudchenko et al. 2015).

Looking further ahead, the combination of these design and fabrication advances may enable even broader RF coverage, including the possibility of tri-band or multi-band SIS receiver architectures. Realising such capabilities will, however, require corresponding progress in ancillary technologies, including ultra-broadband feed horns, wideband local oscillator (LO) sources, and associated quasi-optical components. Although such performance may exceed current technological capabilities, promising approaches exist e.g., ridge-waveguide horn antennas (Pollak and Jones 2018; Flygare et al. 2023; Kooi et al. 2023), which have demonstrated the potential for decade-scale bandwidth operation. Taken together, these developments underscore both the feasibility and the importance of continued innovation in SIS mixer and receiver technologies to realise next-generation ultra-broadband mm/sub-mm observational capabilities.

## Pathway toward large format focal plane array

Traditional high-spectral-resolution observations remain inherently time-intensive, limiting the scope of many key scientific programmes. This constraint is particularly significant for studies requiring large-area coverage at both high spatial and spectral resolution, or for observations complementing wide-field continuum surveys. Increasing mapping speed is therefore a central requirement for next-generation heterodyne instrumentation and can be achieved through the deployment of large-format focal plane arrays. However, current mm/sub-mm heterodyne instruments are typically limited in pixel count, restricting their survey efficiency.

There is thus a pressing need for ultra-sensitive, broadband, large-pixel-count ($>10^{3}$) heterodyne receiver systems. Such capabilities are being actively considered for future facilities, including large-aperture single-dish telescopes such as AtLAST and LST, as well as upgrades to ALMA and next-generation FIR space missions. These instruments are expected to play a critical role in delivering the scientific priorities outlined in major strategic roadmaps, including the US Decadal Survey (of Sciences Engineering NA, Medicine 2023) and the European ASTRONET framework (Astronet 2023). Moreover, they will operate in synergy with forthcoming facilities such as the Square Kilometre Array (SKA, Braun et al. 2015), the Extremely Large Telescope (ELT, Dierickx 2007), SPHEREx (Spectro-Photometer for the History of the Universe, Epoch of Reionization, and ices Explorer, Bock et al. 2026), the Legacy Survey of Space and Time (LSST, Abell et al. 2009), and eROSITA (extended ROentgen Survey with an Imaging Telescope Array, Predehl et al. 2021).

### Scientific motivation for large heterodyne array

Wide-field spectroscopic surveys of the mm/sub-mm sky provide a powerful probe of galaxy evolution from cosmic dawn to the present epoch, enabling studies of baryonic cycling between galaxies and their environments. A key limitation of current facilities is the trade-off between spatial resolution and survey efficiency. While interferometers such as ALMA deliver exceptional sensitivity and angular resolution, their limited field of view restricts wide-area surveys, resulting in incomplete sky coverage and inhomogeneous datasets. Large-format heterodyne FPAs offer a pathway to overcome these limitations by significantly increasing mapping speed while preserving spectral and spatial resolution, enabling homogeneous, large-scale surveys.

A comprehensive sub-mm spectroscopic survey with high sensitivity and broad spectral coverage would enable observations of the first galaxies, probing reionization and large-scale structure formation. Such surveys allow redshift determination and characterisation of gas and dust properties across large galaxy samples, supporting studies of clustering and obscured star formation. Observations of galaxy clusters, including their thermal properties and the relativistic Sunyaev–Zel’dovich effect, further provide constraints on cosmological parameters.

In the nearby Universe, high-resolution spectroscopic mapping enables detailed studies of molecular gas, dust, magnetic fields, and star formation processes within the Milky Way and nearby galaxies. These observations are essential for understanding the multi-phase interstellar medium (ISM) and its connection to the diffuse circum-galactic medium (CGM), which remains poorly characterised due to its low surface brightness. Large-format arrays are uniquely suited to address these challenges through sensitive, wide-field mapping.

More broadly, advancing our understanding of star formation and ISM physics requires large, unbiased samples spanning diverse environments and multiple molecular tracers. Current studies, including those of proto-planetary discs and early star formation, remain limited by sample size. The increased mapping speed and sensitivity of heterodyne FPAs are therefore critical for enabling statistically robust investigations.

The scientific drivers outlined above represent only a subset of the potential applications of large-format heterodyne arrays. Realising these scientific goals requires significant technological advances, including:

- demonstration of quantum-noise-limited sensitivity in array configurations while retaining ultra-broad RF and IF bandwidth (see Sect. 2); and
- development of scalable architectures enabling ultra-large-format FPAs ($>10^{3}$ pixels) with performance comparable to current state-of-the-art instruments.

Achieving these objectives necessitates coordinated progress across multiple domains, including mm/sub-mm optical design (e.g., scalable feedhorn arrays), ultra-low-noise superconducting electronics such as broadband, low-power cryogenic low-noise amplifiers (LNAs), potentially in the form of quantum-limited broadband travelling-wave parametric amplifiers,^6^ and RF & IF signal multiplexing techniques capable of supporting wide bandwidths across large pixel counts (100–1000 elements and beyond) array.

In addition, it is highly desirable for future FPAs to incorporate dual-polarisation sensitivity and, where feasible, balanced or sideband-separating (2SB) capabilities. The combination of ultra-broad RF and IF bandwidths with kilo-pixel-scale, dual-polarisation, and spectrally selective architectures represents a formidable technical challenge, but also a transformative opportunity for mm/sub-mm astronomy.

In the following sections, we outline potential pathways towards realising such systems. While the ultimate objective is the development of ultra-broadband, dual-polarisation, large-format FPAs with advanced spectroscopic capabilities, it is important to emphasise that even partial realisation of these goals would already constitute a substantial advance over current instrumentation and enable significant new scientific discovery.

### Challenges in building a heterodyne array

It is important to recognise that even single-pixel heterodyne receivers are inherently more complex than their bolometric counterparts. Transitioning from single-pixel receivers to large-format heterodyne arrays introduces substantially greater complexity and requires coordinated advances across multiple areas of technology.

The most widely used heterodyne detectors, namely SIS and hot-electron bolometer (HEB) mixers, are typically operated in double-sideband (DSB) mode, in which signal contributions from the upper and lower sidebands (USB and LSB) are superimposed and cannot be distinguished. This limitation can be mitigated through sideband-separating (2SB) architectures, which internally separate the USB and LSB, thereby effectively doubling the usable RF bandwidth compared to DSB operation. Furthermore, when combined with dual-polarisation detection, such systems enable full recovery of the incident electromagnetic power and provide access to the polarisation properties of the incoming radiation, offering valuable insights into phenomena such as magnetic field structures in the interstellar medium.

At present, most operational mm/sub-mm SIS receivers are single-pixel, dual-polarisation 2SB systems. Such configurations typically require four DSB mixer units, together with an orthomode transducer (OMT) and two waveguide hybrid couplers to realise sideband separation. Scaling this already complex architecture to arrays comprising hundreds or thousands of pixels presents a formidable challenge. Over the past two decades, several efforts have been made to develop focal plane arrays, some aiming at large pixel-count, single-polarisation DSB systems (Hughes et al. 2025), while others smaller-scale dual-polarisation 2SB arrays (Daghestani et al. 2024). A short list of other exemplary FPAs developed can be found in Garrett (2018). However, none have yet demonstrated the combination of pixel count, bandwidth, and sensitivity envisaged for next-generation instruments e.g., those for AtLAST, LST, ALMA-2040, and future FIR space missions.

Even when considering the simplest singly-polarised DSB receiver architecture, several key challenges must be addressed:

- *LO distribution:* Efficient and uniform LO delivery across a large array remains a major challenge. Existing approaches, including meandered beam splitters (Buckle et al. 2009), and LO beam multiplexing schemes (Groppi et al. 2010), are often bulky, exhibit non-uniform power distribution, or are complex to implement.
- *Feedhorn fabrication:* Conventional corrugated horns are difficult to manufacture in large numbers at sub-mm and THz frequencies. Promising alternatives include direct-drilled smooth-walled horns (Tan et al. 2012; Leech et al. 2011) and silicon platelet-based technologies (Hu et al. 2021), which offer improved scalability.
- *Interconnect complexity:* Large arrays require substantial numbers of DC bias lines and microwave interconnects, increasing system complexity, thermal load, and potential points of failure.
- *Magnetic biasing:* Each SIS mixer requires magnetic biasing to suppress the Josephson current. Implementing uniform and stable magnetic fields across a large array presents both design and integration challenges.
- *Cryogenic operation:* The need to operate at cryogenic temperatures is compounded in large arrays by the increased hardware volume, wiring density, and associated thermal loads, placing stringent demands on cryostat design and cooling capacity.

**Fig. 5:**
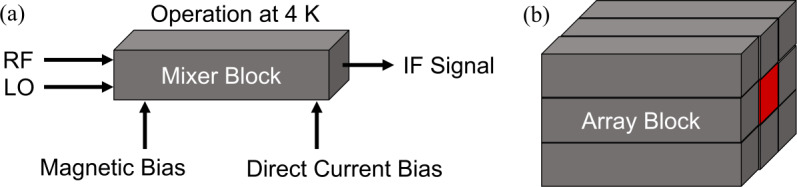
(a) Schematic illustrating the required interfaces for a simple single-ended DSB mixer. (b) Conceptual diagram showing that in a 3×3 array, the central pixel has only two available interfaces for the required interconnections

These challenges are further illustrated by considering the interface requirements of even a single-pixel, singly-polarised DSB SIS receiver, as shown in Fig. 5. Such a system typically requires at least five interfaces: two for RF and LO coupling, one for the IF output, and two for DC connections (biasing and magnetic field control). When extended to a multi-pixel array, spatial constraints quickly become prohibitive. For example, in a simple 3×3 array, the central pixel has limited physical access for routing these interfaces, highlighting the difficulty of scaling conventional architectures. Addressing these challenges will require innovative solutions in receiver architecture, packaging, and system integration. Crucially, any scalable approach must strive to preserve the key performance advantages of single-pixel receivers i.e., ultra-broad RF and IF bandwidths and near-quantum-limited sensitivity, while enabling practical implementation at large pixel counts.

### Large format compact array architecture considerations

The design of a large-format focal plane array (FPA) necessitates the coordinated development of several key technological components. These include, in particular:

1. *OMT:* As discussed previously, polarisation separation can be achieved using waveguide-based structures or quasi-optical (free-space) polarisers. In the context of large-format arrays, however, planar circuit implementations offer a more compact and scalable solution. Accordingly, the approaches considered in this work prioritise on-chip or planar integration to minimise receiver footprint.
2. *LO distribution:* The LO distribution network represents one of the most challenging aspects of FPA design, as it largely dictates the overall system architecture. Conventional approaches include waveguide-based splitter and coupler networks (Hughes et al. 2025), quasi-optical beam-splitting arrangements (Buckle et al. 2009), and optical multiplexing schemes that distribute a single LO source across multiple pixels (e.g. Groppi et al. 2010). Each method presents trade-offs in terms of complexity, uniformity, scalability, and physical footprint. In this work, we focus on planar-circuit-based approaches, which offer potential advantages in compactness and integration.
3. *Array functionality:* The choice of receiver architecture i.e., whether single- or dual-polarisation, and whether operating in DSB, balanced, single-sideband (SSB), or 2SB modes—has significant implications for both LO distribution and the requirement for additional components such as OMTs and hybrid couplers. In this manuscript, we consider the most ambitious yet scientifically compelling configuration: a dual-polarisation 2SB FPA.
4. *Ancillary systems:* Beyond the mixer elements themselves, a fully functional FPA requires a range of supporting technologies, including DC and magnetic biasing networks, feedhorn array design and spacing, mechanical structures optimised for thermal and electromagnetic performance, integration of cryogenic low-noise amplifiers (LNAs), and suitable broadband, low-power IF spectrometers. While these aspects are critical to system performance, their detailed treatment lies beyond the scope of the present work.

**Fig. 6:**
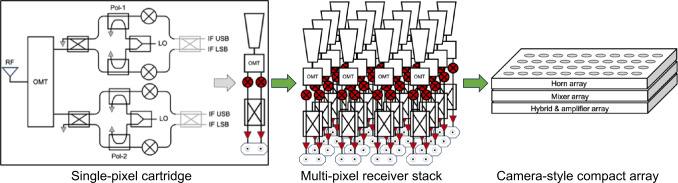
Conceptual evolution from a single-pixel dual-polarisation 2SB mixer to a laterally integrated focal plane array, and ultimately to the proposed vertically integrated FPA architecture

Given these considerations, it is essential to establish a general architectural framework for FPA implementation, independent of specific design choices. Figure 6 illustrates a conceptual evolution from single-pixel heterodyne receivers to architectures capable of supporting arrays comprising hundreds to thousands of SIS mixer elements. For reference, Fig. 6(a) depicts a conventional dual-polarisation 2SB single-pixel receiver, highlighting the additional components required relative to a basic single-ended DSB configuration. This architecture forms the basis of most existing high-performance heterodyne receivers.

To date, the majority of FPA development efforts have focused on replicating such single-pixel receiver architectures in a lateral arrangement to form multi-pixel arrays. In practice, the complexity of the full 2SB dual-polarisation configuration often necessitates simplifications or compromise, such as reducing to single-polarisation or DSB operation, or limiting the overall pixel count. These approaches typically aim to minimise footprint by compactly arranging multiple single-pixel receiver modules, as illustrated in Fig. 6(b). While this methodology is viable for modest array sizes (e.g., 16–25 pixels, and in some cases up to ∼100 pixels), it becomes increasingly difficult to scale beyond this range due to constraints in packaging, interconnect density, and LO distribution.

A potential step-change in array architecture is therefore required. In this work, we propose a transition from lateral integration to a vertically integrated architecture, analogous to approaches employed in bolometric imaging arrays, as illustrated in Fig. 6(c). In the following sections, we outline a conceptual framework for implementing such architectures.

### Sidebands-separating mixers

**Fig. 7:**
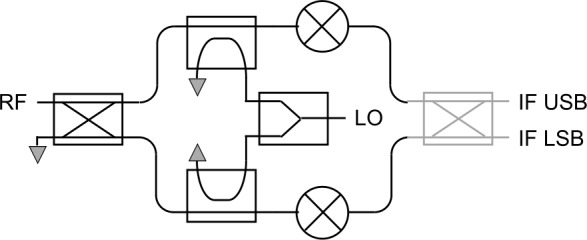
Schematic diagram illustrating the components required to realise a 2SB mixer, including a pair of DSB mixers, directional couplers, an in-phase power divider, an RF quadrature hybrid, and an IF quadrature hybrid. In this example, the in-phase power divider is implemented in the LO injection path and the RF quadrature hybrid in the RF path; however, the configuration remains valid if these passive components are interchanged between the RF and LO paths

Figure 7 illustrates the fundamental architecture of a sideband-separating (2SB) receiver, designed to isolate the upper sideband (USB; $\omega _{\mathrm{USB}} = \omega _{\mathrm{RF}} - \omega _{ \mathrm{LO}}$) and lower sideband (LSB; $\omega _{\mathrm{LSB}} = \omega _{\mathrm{LO}} - \omega _{ \mathrm{RF}}$) components. In conventional double-sideband (DSB) receivers, these components overlap within the IF band, leading to ambiguity and potential contamination of spectral line measurements. The 2SB architecture mitigates this issue by separating the sidebands prior to detection, thereby enabling cleaner spectral interpretation and effectively doubling the usable RF bandwidth. For this reason, 2SB receivers are widely adopted in modern astronomical facilities, including most ALMA bands.

In addition to the two DSB mixer elements, a conventional 2SB receiver requires several auxiliary RF and IF components, including:

- an in-phase $-3\text{ dB}$ power divider,
- a 90^∘^ hybrid coupler,
- a set of directional couplers, and
- an IF quadrature hybrid.

With the exception of the IF quadrature hybrid, these components^7^ are traditionally implemented using waveguide technology (Khudchenko et al. 2023). While this approach has been successfully realised in existing systems, it relies on complex and bulky waveguide assemblies. Such implementations impose significant constraints on scalability, particularly for large-format focal plane arrays, and limit compatibility with compact or highly integrated receiver architectures (see Sect. 4).

Overcoming these limitations represents a key challenge for next-generation heterodyne instrumentation. In the following, we propose an alternative approach in which the conventional waveguide-based hybrid block, comprising the in-phase power divider, 90^∘^ hybrid, and directional couplers, is replaced by planar superconducting chip-based components. This strategy aims to substantially reduce receiver footprint and enable scalable integration, thereby facilitating the realisation of large-format heterodyne FPAs.

In the following, we shows our proposal to replace these RF waveguide components i.e., the in-phase –3 dB power divider, the 90^∘^ hybrid coupler and the directional coupler with planar on-chip component to minimise the receiver footprint, hence enable construction of large FPAs.

#### 2SB mixers: partial waveguide approach

**Fig. 8:**
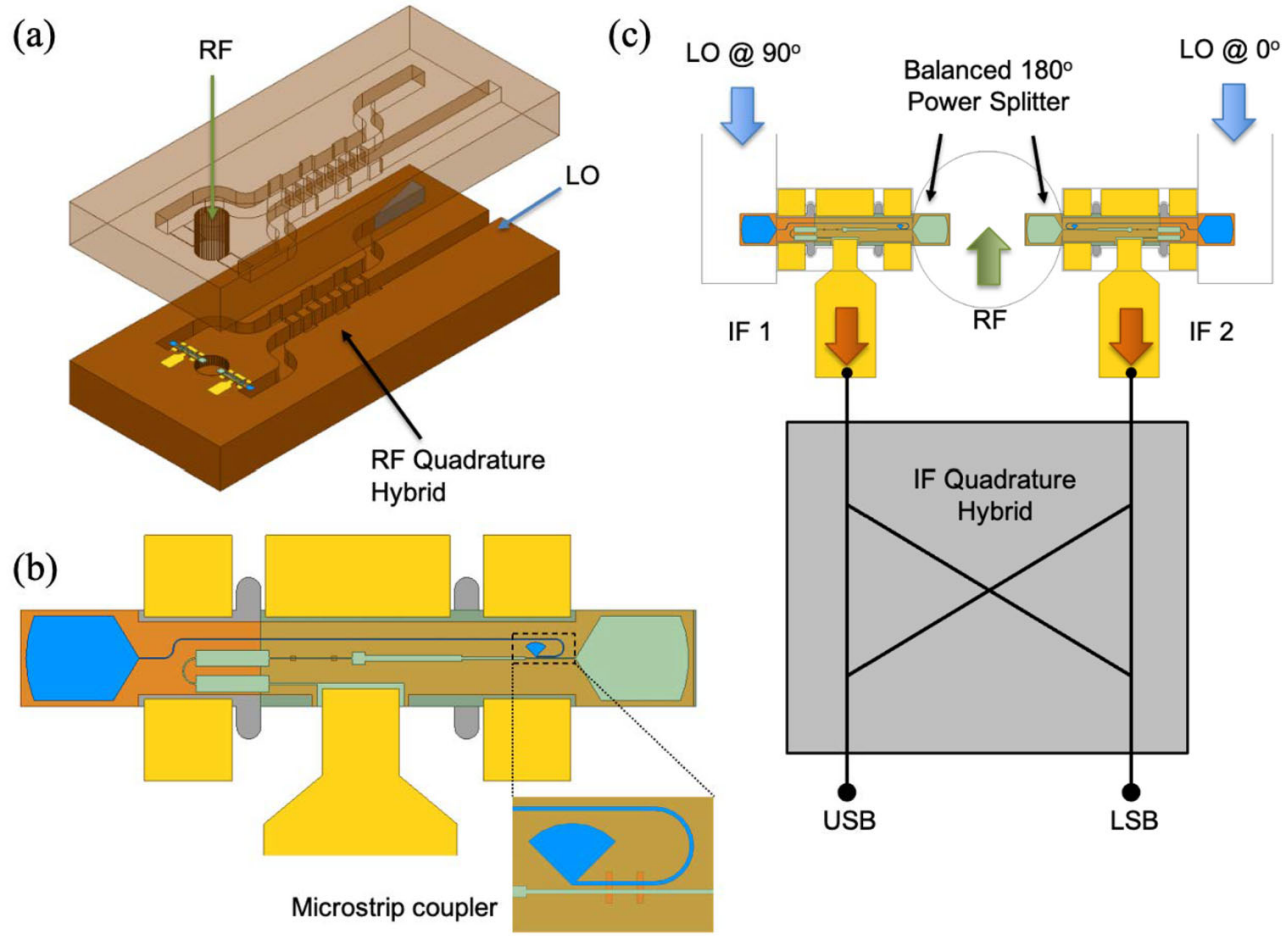
(a) Proposed layout of a partial-waveguide 2SB mixer, comprising a waveguide RF quadrature hybrid and a pair of DSB mixers. (b) Layout of the DSB mixer chip, where green-shaded regions indicate the RF signal path and blue-shaded regions indicate the LO injection path, coupled from the reverse side of the chip via a microstrip coupler located near the RF probe antenna. (c) Top-down schematic of the 2SB mixer, illustrating LO injection with a 90^∘^ phase difference and RF signal splitting; in this configuration, the RF signals are delivered with a relative 180^∘^ phase difference (functionality similar to an in-phase splitter)

Figure 8 illustrates a hybrid architecture in which selected waveguide components of a conventional 2SB receiver are replaced in order to reduce overall system footprint. In this approach, the waveguide-based in-phase $-3\text{ dB}$ power divider and directional couplers are eliminated, while retaining the RF quadrature hybrid as a waveguide component.

The two DSB mixers are arranged in a back-to-back configuration across a circular waveguide, analogous to the geometry discussed in Sect. 2.3. In this configuration, the incident RF and LO signals are naturally split equally between the two mixers, effectively replicating the functionality of a −3, dB power divider without requiring an additional component. It is noted that the two mixers experience a relative phase difference of 180^∘^, rather than being strictly in phase. However, this does not impair 2SB operation, provided that the RF and LO signals delivered to the mixers maintain the required 90^∘^ phase relationship for proper sideband separation.

To further reduce system complexity, the LO injection mechanism is implemented using an on-chip planar microstrip coupler (Tan and Yassin 2017b). In this scheme, the LO signal is introduced from the reverse side of the mixer chip, propagating towards the RF input region where it is coupled into the signal path via the microstrip structure. The feasibility of such planar LO coupling schemes has been demonstrated experimentally (Monje et al. 2007).

This architecture significantly reduces the reliance on bulky waveguide components by effectively eliminating much of the conventional hybrid block. Most of the RF functionality is either integrated on-chip or achieved through strategic placement of the mixer elements except the RF quadrature hybrid. As such, this partial waveguide approach represents an intermediate step towards fully integrated, planar 2SB receiver architectures, offering a favourable balance between performance, compactness, and scalability.

#### 2SB mixers: minimal waveguide approach

**Fig. 9:**
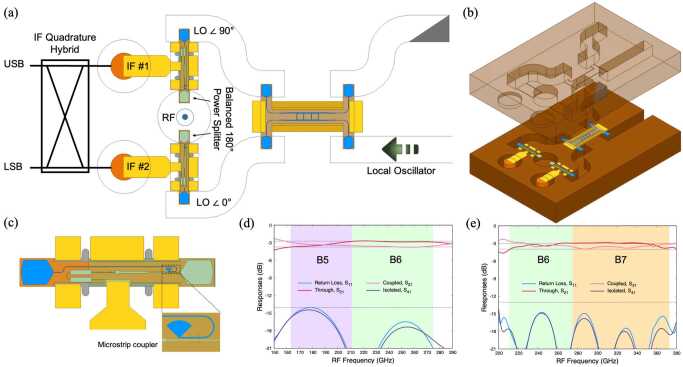
(a) Top-down schematic and (b) three-dimensional view of the proposed minimal-waveguide 2SB mixer architecture. (c) Corresponding DSB mixer design, illustrating that the same mixer configuration can be employed in this approach. (d) Simulated performance of a planar on-chip RF hybrid covering ALMA Bands 5+6, obtained using HFSS. (e) As in (d), but for a planar superconducting RF hybrid operating across Bands 6+7

The approach described above can be further refined to reduce the receiver block footprint by replacing the remaining waveguide-based RF quadrature hybrid with a planar, on-chip implementation. An example of this configuration is shown in Fig. 9(a) and (b). In this architecture, the RF quadrature hybrid is realised on a separate superconducting chip, employing a silicon-on-insulator (SoI) substrate and niobium (Nb) microstrip circuitry to minimise transmission losses. The LO injection is implemented using the same planar microstrip coupler described previously (Fig. 9(c)). Interconnection between the hybrid chip and the DSB mixer chips is achieved via short waveguide sections, thereby retaining a minimal level of waveguide integration while significantly simplifying the overall structure.

The simulated performance of the superconducting hybrid chip is shown in Fig. 9(d) and (e), for operation across frequency ranges corresponding to ALMA Bands 5–6 and Bands 6–7, respectively. The results indicate that the hybrid achieves better than $-13\text{ dB}$ return loss and isolation across the operating bands, while maintaining amplitude imbalance within approximately 1 dB between the through and coupled ports.

This minimal waveguide approach further reduces the reliance on bulky waveguide components compared to the partial waveguide configuration, while preserving the essential phase and amplitude characteristics required for 2SB operation. As such, it represents a key step towards fully planar, highly integrated 2SB receiver architectures suitable for large-format focal plane arrays.

#### 2SB mixers: planar on-chip integration approach

**Fig. 10:**
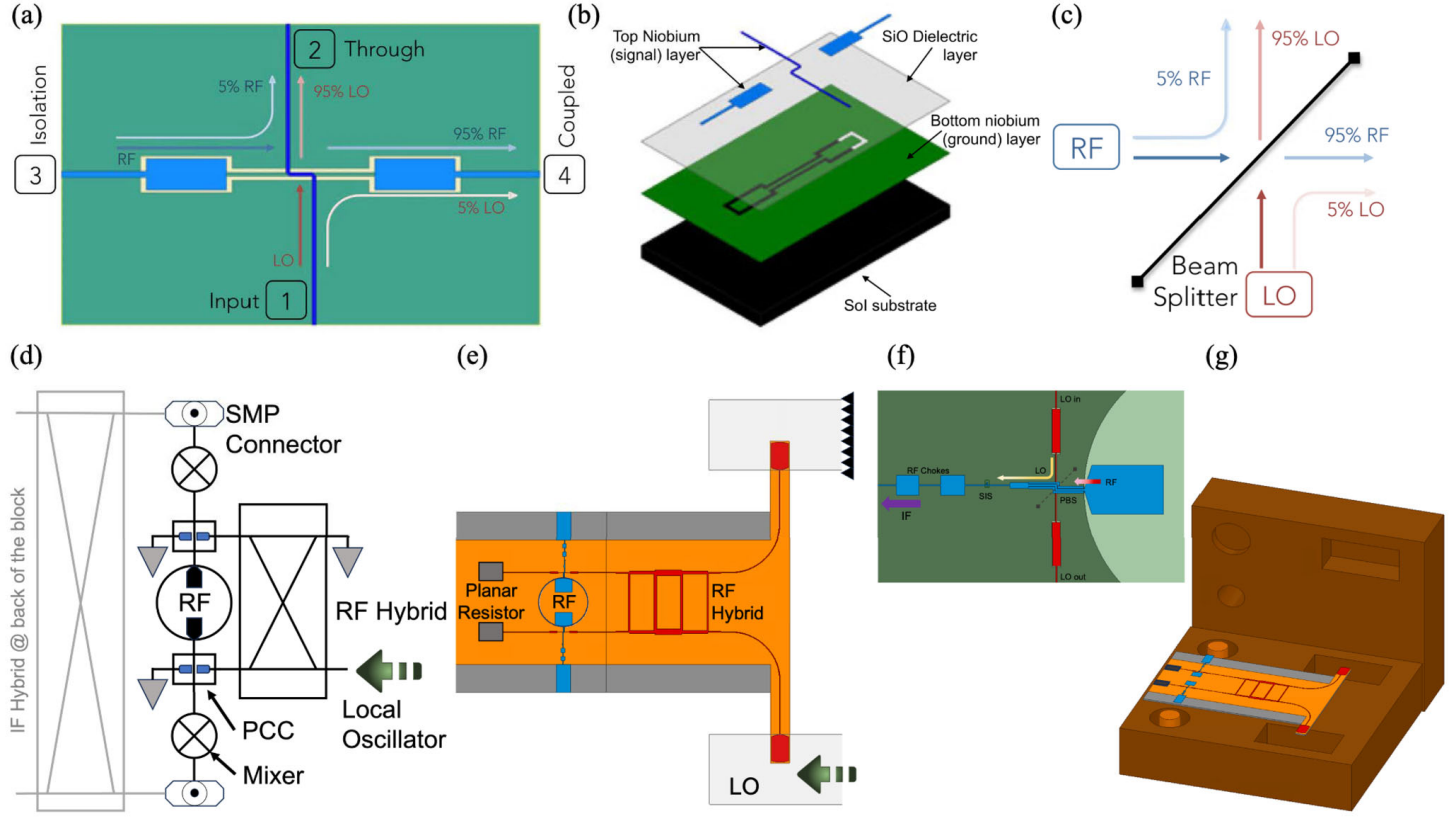
(a) Top-down and (b) three-dimensional stack-up view of the planar cross-coupler, implemented using Nb ground and wiring layers separated by an SiO dielectric on an SoI substrate. (c) Transmission and reflection characteristics of a free-space beam splitter, illustrating the analogous behaviour of the planar cross-coupler. (d) Schematic of a fully planar, on-chip integrated 2SB mixer incorporating the cross-coupler. (e) Top-down layout of the planar 2SB mixer chip. (f) Enlarged view of the DSB mixer coupled to the planar cross-coupler. (g) Three-dimensional representation showing the integration of the planar 2SB mixer chip within a split-block waveguide structure

The minimal-waveguide 2SB mixer architectures described above achieve a compact receiver footprint; however, their scalability to large-format arrays remains limited. A potential pathway to overcome this limitation is to replace the conventional microstrip coupler with an alternative planar coupling scheme based on an innovative cross-coupler architecture (Tan and Yassin 2017a, 2018; Tan et al. 2019). Figure 10(a)–(c) illustrate the structure and operating principle of the planar cross-coupler. This device exhibits functionality analogous to a quasi-optical free-space beam splitter, which is widely used in mm/sub-mm array receivers. Detailed descriptions of its operating principles and measured performance can be found in Tan and Yassin (2017b).

In essence, the planar cross-coupler can be viewed as an evolution of a planar cross-over structure (Abbosh et al. 2012). It comprises two transmission lines, typically a coplanar waveguide (CPW) and a microstrip, arranged to intersect with minimal undesired interaction. In the conventional cross-over, the lines are orthogonal to minimise coupling. In contrast, the cross-coupler introduces a controlled interaction by locally aligning a section of the microstrip parallel to the CPW, enabling a defined fraction of power to be transferred between the two lines. As shown in Fig. 10(a) and (c), the resulting power-splitting behaviour closely mimics that of a free-space beam splitter.

An important advantage of this approach is its compatibility with the multilayer superconducting transmission line technology already employed in SIS mixer fabrication (i.e., ground-plane/dielectric/wiring layer). Consequently, the planar cross-coupler can be readily integrated into existing fabrication processes, providing a compact alternative to conventional quasi-optical or waveguide-based beam-splitting elements.

The incorporation of the planar cross-coupler enables a significant simplification of the 2SB receiver architecture. As illustrated in Fig. 10(d)–(f), the RF quadrature hybrid is now fully integrated onto the same substrate as the DSB mixer elements. This eliminates the need for interconnecting waveguide sections and replaces the microstrip-coupler LO coupling scheme, again further simplify the receiver chips’ layout.

The resulting architecture is a fully planar, on-chip integrated 2SB mixer, in which all RF signal routing and LO coupling functions are realised within the superconducting circuit on a single chip. The only remaining waveguide interfaces are those required for RF signal input, LO injection, and termination of the quadrature hybrid. Compared to the partial waveguide approach (Fig. 8), this configuration offers a substantially reduced footprint, potentially by an order of magnitude, while maintaining the functional requirements for sideband separation. Such compact architecture could reduce the footprint of existing 2SB receivers, thereby allows for incorporating more multi-band receivers on an existing telescope, even not for the FPA purposes.

### Extension to singly-polarised 2SB array

Once a fully planar, on-chip integrated 2SB mixer architecture is established, its extension to a singly polarised linear array becomes conceptually straightforward. As described previously, the planar cross-coupler functions analogously to a free-space beam splitter, coupling only a fraction of the LO power to each SIS mixer element. In the single-pixel implementation, the remaining LO power is typically dissipated in an on-chip resistive termination. However, this residual LO power can instead be utilised to drive subsequent mixer elements arranged in an array configuration.

**Fig. 11:**
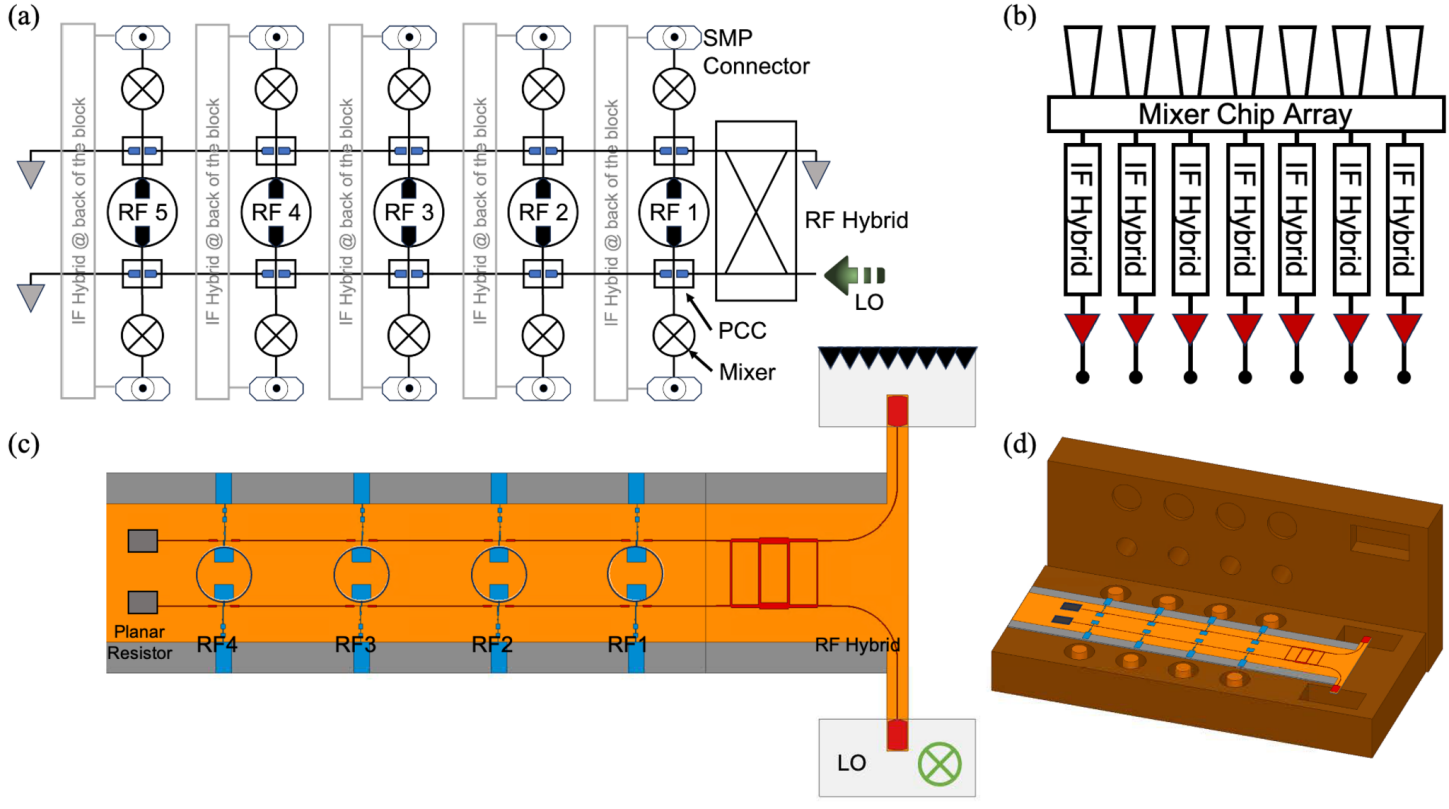
(a) Schematic of an exemplary five-pixel linear (1-D) 2SB mixer array. (b) Cross-sectional schematic of the linear array, including the feedhorn array, mixer chip array, IF hybrid network, and LNAs, illustrating the concept of a horizontally integrated array receiver. (c) Example layout of the planar on-chip circuitry for the linear array. (d) Illustration of the mounting configuration of the linear array chip within a split-block mixer array structure

Figure 11 illustrates this concept. In this architecture, the LO power transmitted through the first mixer stage is progressively coupled to downstream mixer elements via successive planar cross-couplers. By appropriately designing the coupling strength achieved i.e., by tuning the interaction length of the coupling region, it is possible to maintain a relatively uniform LO pumping level across all mixer elements in the array. This approach enables the realisation of a single-chip, singly polarised 2SB linear array, forming the basis for lateral integration of larger-format systems.

One important consideration in this architecture is the physical spacing between array elements. The interconnection of successive mixer units via microstrip transmission lines introduces potential vulnerabilities, including fabrication sensitivity and increased RF losses. While losses in the LO distribution path are generally less critical than those in the RF signal path, they must nevertheless be carefully managed to ensure uniform mixer performance. The optimal pixel spacing is therefore determined by a combination of fabrication constraints, electromagnetic performance, and system-level considerations such as telescope optics and mapping strategy. In practice, the array pitch may be minimised to approach the physical limits set by feedhorn aperture size. Alternatively, quasi-optical re-imaging techniques may be employed to adapt the beam spacing on the sky, allowing for e.g., half-wavelength or larger beam separation while maintaining a compact focal plane layout.

**Fig. 12:**
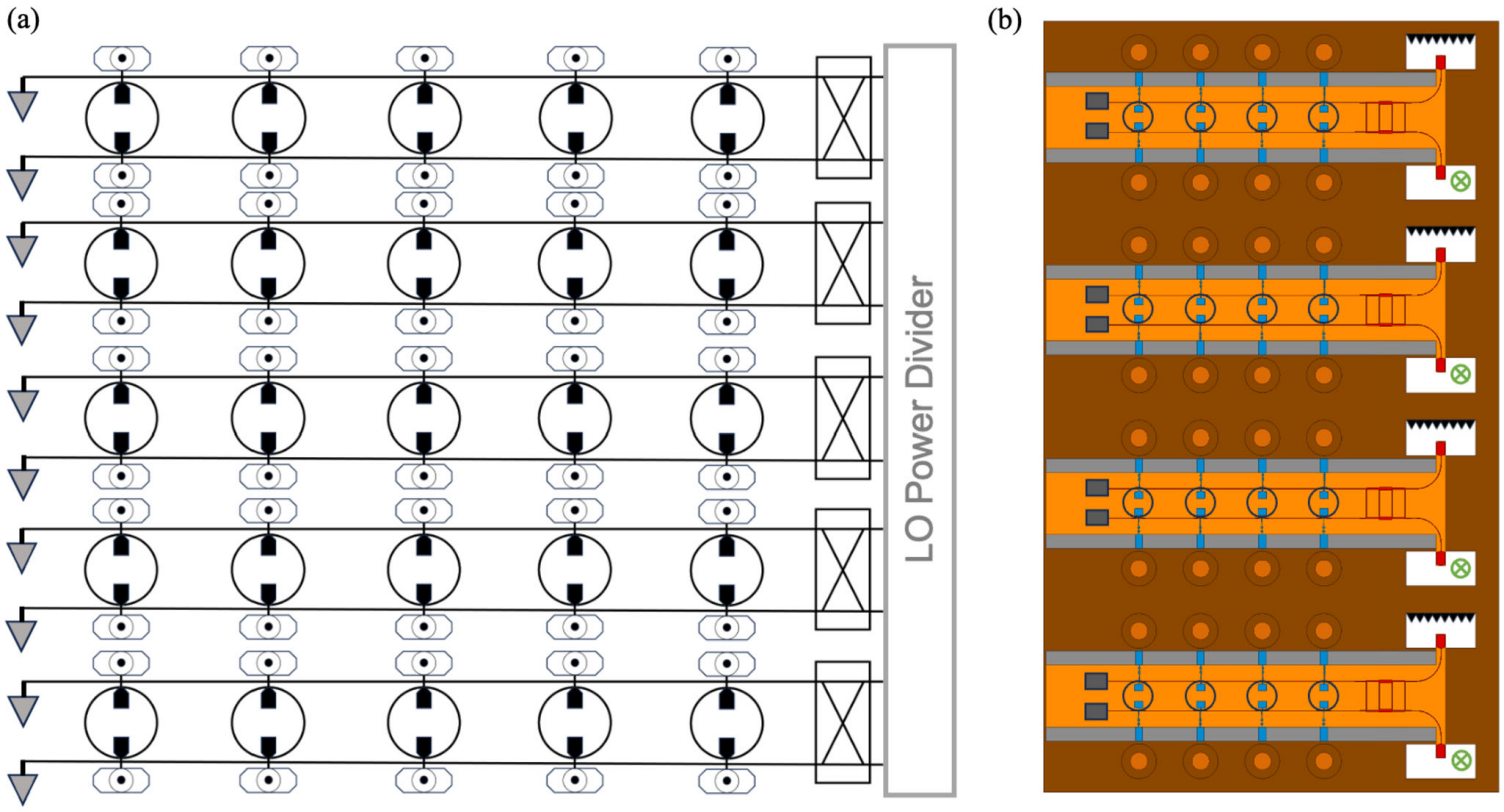
(a) Schematic illustrating the proposed layout of a two-dimensional singly polarised 2SB array. Where sufficient LO power is available, an LO power divider network can be used to distribute the signal across multiple mixer elements. (b) Conceptual illustration of how linear array chips can be combined to realise a laterally integrated FPA architecture

Building on this linear architecture, extension to two-dimensional arrays is a natural progression. As shown schematically in Fig. 12, multiple one-dimensional singly polarised 2SB array chains can be arranged in parallel to form a two-dimensional array. Provided that sufficient LO power is available, the LO signal can be multiplexed and distributed across these parallel channels, enabling simultaneous pumping of the entire array. This results in a laterally integrated two-dimensional heterodyne array architecture with significantly enhanced mapping capability.

This approach represents a scalable pathway towards large-format heterodyne FPAs, leveraging planar integration and efficient LO power utilisation. While further optimisation is required particularly in terms of uniformity, loss management, and system integration, it provides a promising foundation for the development of compact, high-performance array receivers.

### Extension to dual-polarisation 2SB array

**Fig. 13:**
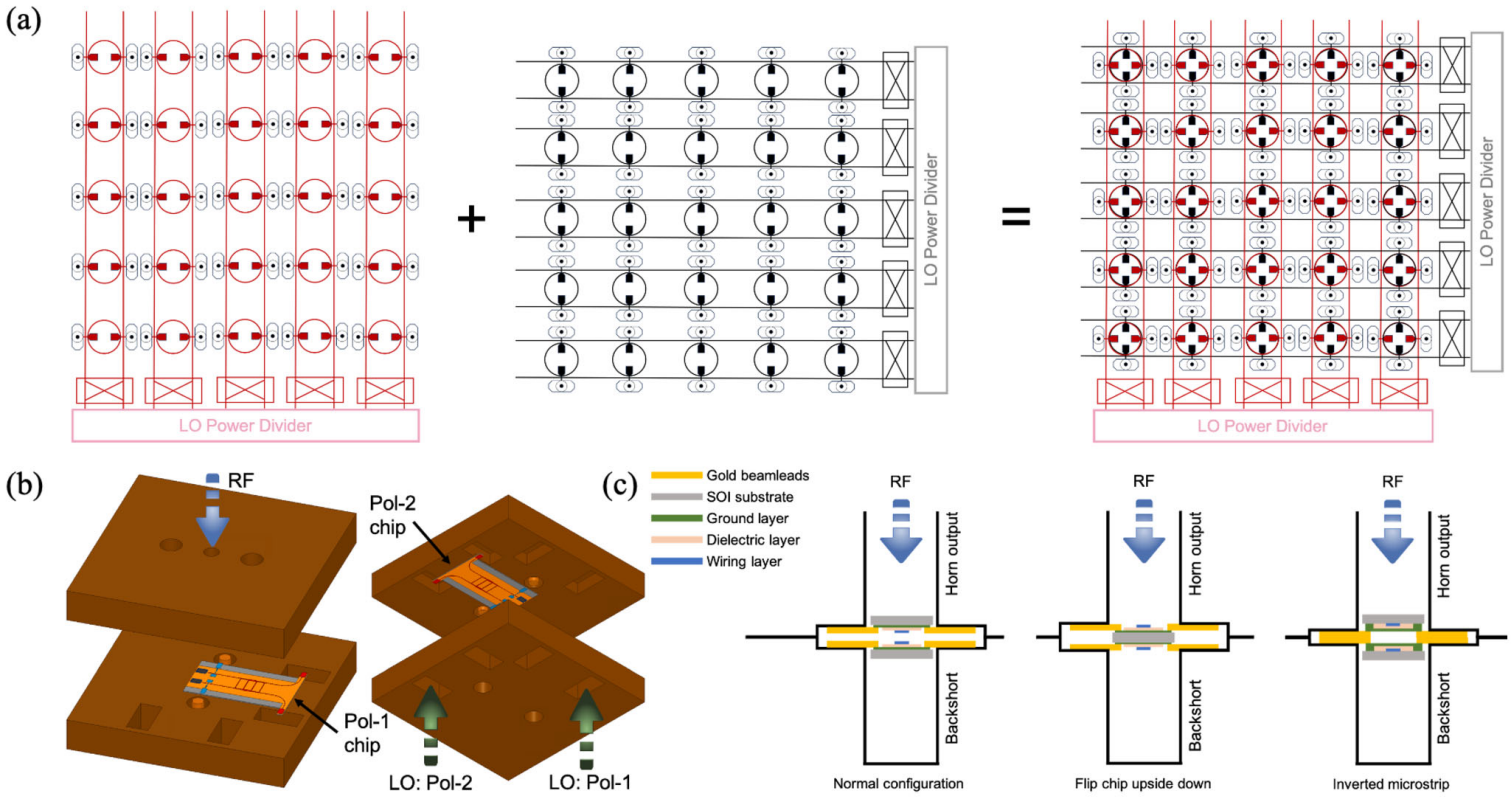
(a) Schematic illustrating how two singly polarised 2SB arrays can be combined to form a dual-polarisation 2SB array. (b) Three-dimensional view showing that each polarisation array chip is mounted separately on the upper and lower halves of a split-block mixer assembly. (c) Illustrations of three possible back-to-back mounting configurations for the array chips. Dimensions in the sketches are exaggerated for clarity: typical trench depths are of order 10 μm, while the backshort depth is on the order of hundreds of microns, depending on the RF band

Building upon the singly polarised two-dimensional 2SB array architecture described above, extension to dual-polarisation operation can be achieved by stacking two such arrays orthogonally. This concept is illustrated schematically in Fig. 13(a), where each layer is sensitive to one linear polarisation component of the incident electromagnetic field.

A key practical consideration in this approach is the precise alignment of the two array chips with respect to the input waveguide. Figure 13(b) and (c) present a proposed implementation, in which one array chip is mounted on the lower block of the receiver structure, analogous to the singly polarised configuration, while the second array is mounted on the upper block. This configuration is enabled by the use of ultra-thin membrane technologies for the mixer substrates, such as SoI or silicon nitride (SiN) membranes, which allow compact stacking with minimal separation between the two layers.

Several mounting strategies can be considered for assembling two beamlead-equipped membrane-based mixer chips in a back-to-back configuration. In the first approach, illustrated in the first panel of Fig. 13(c), the trench accommodating the chips is slightly deepened to house both layers. Provided that the wiring layers of the two chips do not come into direct contact, this configuration remains feasible, as the electromagnetic fields in microstrip circuits are predominantly confined between the signal and ground planes. However, this approach may be less suitable for transmission line implementations based on CPW, stripline, or slotline geometries, where field confinement differs. An alternative and potentially simpler configuration is shown in the second panel of Fig. 13(c), in which both array chips are mounted up-side down i.e., inverted orientation compared to the first option. This allows the two substrates to provide mechanical support to one another, reducing the required trench depth and mitigating the risk of unwanted electromagnetic coupling between the circuits (field lines shielded by ground planes).

In both configurations, variation in trench depth introduces an additional consideration: the effective backshort distance seen by each probe antenna may differ slightly between the two polarisation layers, potentially degrading RF coupling efficiency, although the affected performance is expected to be minimal. The impact of this effect can be evaluated and optimised through detailed three-dimensional electromagnetic simulations. One possible mitigation strategy is to employ a spatially varying backshort geometry tailored to the orthogonal orientations of the two arrays e.g., different quarter of the circular backshort has a different height, although this increases mechanical complexity.

A further refinement involves the use of inverted microstrip architectures, as illustrated in the third panel of Fig. 13(c). In this configuration, the required trench depth is reduced to the thickness of the beam leads (typically 1–2 μm), rather than the full substrate thickness. Although the substrate extends partially into the waveguide and backshort regions, the resulting electromagnetic loading can be accurately accounted for during the design process through numerical simulation.

One notable feature of the array architectures presented in this section is that the RF, LO, and IF interfaces are all located on the upper and lower surfaces of the array (or mixer) block, leaving the four lateral faces unobstructed. This provides an opportunity to incorporate a pair of moderately sized Helmholtz coils on either side of the array block to generate the magnetic field required to suppress the unwanted Josephson current in the SIS junctions. Such an approach is considerably more practical for large-format arrays than employing an individual magnetic bias coil for each mixer element. Its effectiveness, however, relies on achieving a high degree of uniformity in the tunnel junction characteristics across the entire array, allowing all mixers to be biased using a common magnetic field. An alternative solution is to employ miniature permanent magnets positioned adjacent to the tunnel junctions, thereby providing local magnetic biasing without the need for individual electromagnets.

While these approaches require further investigation to assess their practical feasibility, they collectively demonstrate that vertical stacking of orthogonally oriented mixer arrays provides a viable pathway towards dual-polarisation operation in large-format heterodyne arrays. Importantly, these concepts remain compatible with current fabrication and micro-machining technologies.

**Fig. 14:**
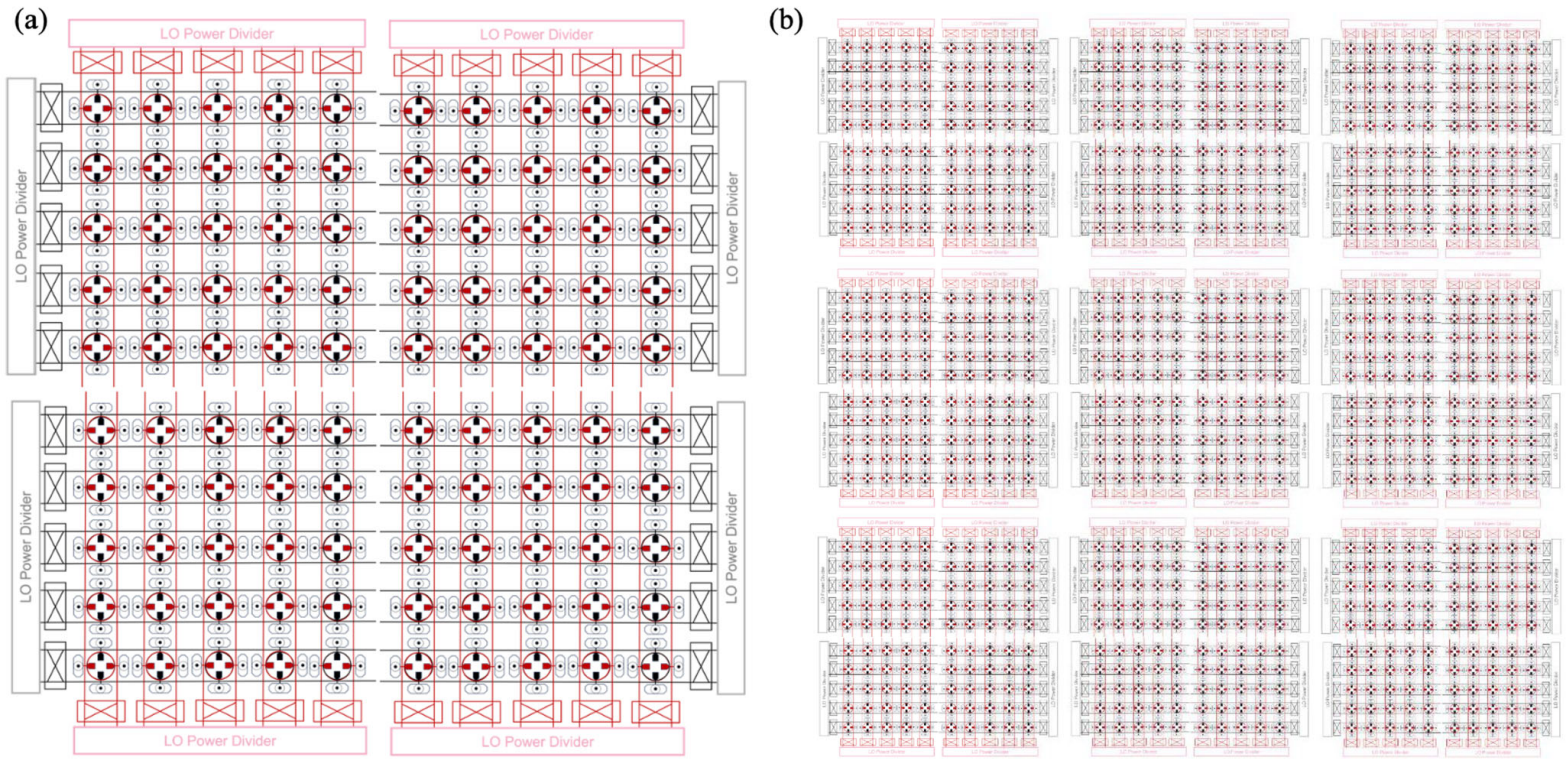
(a) Schematic illustrating the extension of a 5×5 array to a 10×10 array. (b) Further scaling of the 100-pixel dual-polarisation 2SB array to a 900-pixel array, equivalent to 3600 singly polarised DSB mixer elements

Once a 2-D dual-polarised 2SB array has been realised, scaling to larger array formats becomes a matter of modular extension. As illustrated in Fig. 14(a), a 5×5 array (25 pixels) can be extended laterally to form a 10×10 array (100 pixels). Notably, such a system is functionally equivalent to a 400-pixel singly polarised DSB array in terms of detector count. This modular approach can be extended further, as shown in Fig. 14(b), where arrays comprising several hundred pixels (e.g., 900 pixels) are envisaged. This corresponds to an effective detector count of 3600 singly polarised DSB elements. Such bolometric camera-style architectures align with the horizontal integration strategy discussed in Sect. 3.3, and represent a viable pathway towards kilo-pixel-scale heterodyne arrays required for next-generation facilities such as AtLAST, and LST.

### Short summary

In this section, we have outlined a potential pathway towards the realisation of kilo-pixel SIS mixer-based heterodyne focal plane arrays, including the key technical challenges that must be addressed to achieve such advanced systems with dual-polarisation sensitivity and sideband-separating capability. If successfully implemented, these architectures would significantly enhance the observational performance of current single-pixel heterodyne receivers. Future facilities, including ALMA 2030/2040 and AtLAST/LST, are expected to require such capabilities to fully realise their scientific potential. It is important to recognise that even partial realisation of these concepts would already constitute a major advance. For example, a singly polarised 2SB FPA with large pixel count and compact architecture would deliver substantial improvements in mapping speed and survey efficiency, and would be sufficient to enable transformative science in the mm/sub-mm regime.

Looking beyond the proposed dual-polarisation 2SB FPA architecture, further enhancements remain possible. In particular, balanced 2SB receiver configurations offer the potential to reduce receiver noise temperature by suppressing LO-induced noise contributions. The concepts presented here could, in principle, be extended to realise compact, large-format dual-polarisation balanced-2SB FPAs. In addition, alternative heterodyne receiver architectures, such as correlation receivers (Kooi 2008), may be adapted within the same framework to address specific observational requirements.

Further advances in enabling technologies, including ultra-compact, ultra-low-power cryogenic IF amplifiers with integrated bias-tees (Montazeri et al. 2015; Bardin 2021) and digital implementations of IF quadrature hybrids (Finger et al. 2015; Rodriguez et al. 2018), have the potential to significantly simplify the proposed receiver architectures, reducing system complexity while further improving their scalability towards kilo-pixel heterodyne arrays.

More broadly, this work demonstrates that advanced heterodyne receiver functionalities, including dual-polarisation detection, sideband separation, balanced operation, and correlation modes, can be achieved through the integration of RF components using superconducting planar circuit technologies. Continued progress in key enabling technologies, such as higher critical current density SIS junctions, sub-micron fabrication processes, and low-loss high-gap superconducting materials (e.g., NbTiN or NbN) for operation beyond the niobium gap frequency, will further enhance performance. Combined with ultra-thin membrane substrate technologies and beam-lead interconnects, these developments form the foundation for next-generation heterodyne FPAs. Together, they offer a realistic pathway towards achieving orders-of-magnitude improvements in observing capability relative to current instruments, thereby enabling a new era of high-throughput, high-fidelity mm/sub-mm spectroscopy.

## Simultaneous observing multi-band receivers (SOMBRs)

As discussed in Sect. 1, SIS receivers play a critical role in very long baseline interferometry (VLBI) observations, including efforts to image the event horizon of black holes with the Event Horizon Telescope (EHT). The EHT achieves this by synthesising a global array of mm/sub-mm observatories to form an Earth-sized interferometer. Ongoing developments aim to further extend the interferometric baseline through the inclusion of space-based elements, such as the proposed Black Hole Explorer (BHEX, Marrone et al. 2024; Tong et al. 2025, 2024) and the Space-based High-resolution Array for Radio Astronomy and Physics (SHARP, Villard et al. 2026) missions, among others.

For black hole imaging, simultaneous multi-band observations are essential for capturing the rapidly evolving emission structures in the vicinity of the event horizon (Backes et al. 2017). In addition, concurrent observations across multiple frequency bands enable phase-transfer calibration techniques, whereby phase information measured at lower frequencies, where atmospheric effects are less critical, is used to stabilise higher-frequency interferometric observations. Simultaneous multi-band capability also facilitates the observation of multiple spectral features within a single coordinated campaign, thereby enhancing scientific return.

These requirements introduce both new opportunities and challenges for receiver design, motivating the development of innovative architectures capable of simultaneous multi-band operation. In this section, we introduce the concept of a simultaneous observing multi-band receiver (SOMBR), based on a cryogenic diplexer operating at ∼4 K, while retaining compatibility with sideband-separating heterodyne operation.

### Existing simultaneous observing scheme

While many space-borne heterodyne instruments support simultaneous multi-band observations through the use of free-space diplexing optics, ground-based systems typically operate in single-band mode. This limitation arises primarily because free-space diplexers, when implemented in ground-based receivers, are generally located at ambient temperature stages and introduce non-negligible insertion losses. As pre-mixer optical components, these losses directly translate into increased system noise, thereby degrading the near-quantum-limited sensitivity of modern SIS receivers. For example, a 5% optical loss preceding the mixer corresponds to an effective noise contribution of approximately $300 \times 0.05 = 15\text{ K}$. At 1 mm wavelength, where the DSB quantum noise limit is of order 30 K, this additional noise represents a ∼50% increase. Furthermore, the associated signal attenuation propagates through the receiver chain, compounding the degradation in sensitivity. Consequently, the use of warm optical diplexers has remained unattractive for high-sensitivity ground-based heterodyne systems.

An alternative approach has been demonstrated by Masui et al. (2020, 2021) for the Osaka 1.85 m mm/sub-mm telescope, in which simultaneous multi-band operation was achieved using waveguide-based diplexing components. In this implementation, a broadband RF signal is first divided into lower- and higher-frequency bands using a waveguide diplexer comprising a pair of broadband quadrature hybrids combined with high-pass filters. Each branch is subsequently further subdivided using additional diplexer stages, enabling simultaneous observation of multiple spectral lines.

By cascading three such waveguide diplexers, the system partitions the RF bandwidth into four discrete sub-bands matched to the broad IF bandwidth of the SIS mixers. This architecture enables simultaneous detection of four spectral lines, albeit within fixed frequency ranges. As a consequence of the diplexing scheme, the receiver operates in single-sideband (SSB) mode, with the LO frequency chosen such that the desired upper and lower sidebands fall within the predefined filter passbands.

**Fig. 15:**
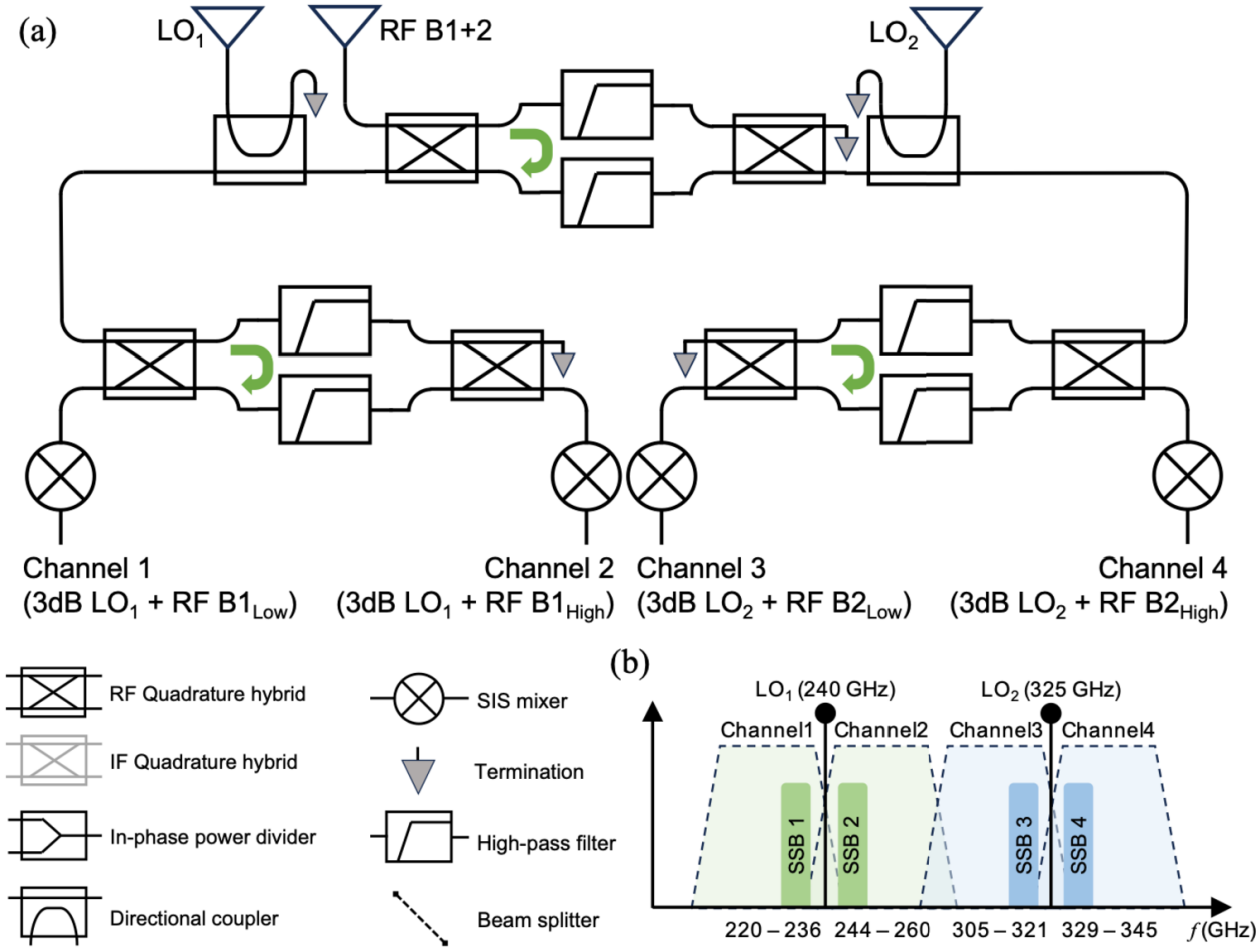
(a) Schematic illustrating the waveguide diplexing-based quad-band (fixed-band) SOMBR demonstrated by Masui et al. (2021), reproduced here for reference. (b) Illustration of the four diplexed RF bands and the corresponding narrower detected spectral ranges determined by the fixed LO frequencies

As illustrated in Fig. 15, this approach requires a substantial number of components, including six quadrature hybrids, six filter elements, four SIS mixers, two directional couplers for LO injection, and multiple broadband antennas. The waveguide diplexers and filters are realised using high-aspect-ratio structures (typically ∼10:1), demanding extremely precise machining. While this represents an impressive technical achievement, such fabrication capabilities and associated expertise are not widely accessible, potentially limiting broader adoption.

In the following sections, we propose a conceptually similar, yet significantly simplified, approach with two key enhancements:

- *Frequency agility:* The fixed-band constraint is removed by enabling continuous RF tuning via conventional LO control, while simultaneously retaining sideband-separating operation in each observing band. This allows full utilisation of the RF bandwidth, rather than restricting observations to predefined frequency windows.
- *Planar integration:* The waveguide diplexers are replaced with superconducting planar circuit components, reducing insertion loss and substantially minimising system footprint. In this approach, filtering functions can be implemented using a choice of simple rectangular waveguide sections (e.g., high-pass behaviour) and planar superconducting filters (e.g., low-pass responses), as discussed in subsequent sections.

As will be demonstrated, this architecture significantly reduces the number of required components and leads to a more compact and scalable implementation. In essence, the resulting SOMBR configuration resembles a cascade of 2SB receiver modules, enabling multi-band simultaneous observation with minimal additional complexity. If successfully realised, this concept could be extended to provide continuous, simultaneous coverage across the mm/sub-mm spectrum, and potentially into the supra-THz regime by incorporating hot-electron bolometer (HEB) receivers in place of SIS mixers, without requiring complex and bulky diplexing assemblies.

### Proposed dual-band SOMBR scheme

As introduced in Fig. 7, a conventional 2SB SIS mixer architecture differs from the simpler DSB configuration by incorporating additional RF and IF circuitry to separate the upper and lower sidebands. Specifically, the incoming RF signal is split by an RF quadrature hybrid into two equal-amplitude components with a 90^∘^ phase difference while the LO signal is injected in phase, into a pair of DSB mixers.^8^ The LO is typically distributed via identical directional couplers. The resulting IF outputs are then recombined using an IF quadrature hybrid, enabling separation of the upper and lower sidebands into distinct IF channels.

In this section, we demonstrate how two such 2SB mixer units can be combined to enable dual-band simultaneous observation by incorporating only a minimal number of additional components. In our implementation, we employ high-pass filters (HPFs) to partition the RF signal into two frequency bands. While low-pass or band-pass filters could also be used, HPFs are particularly attractive due to their simplicity, as they can be realised using straight rectangular waveguide sections with appropriately chosen cutoff dimensions. The filtering performance may be further enhanced using high-aspect-ratio structures, as demonstrated in Masui et al. (2021).

**Fig. 16:**
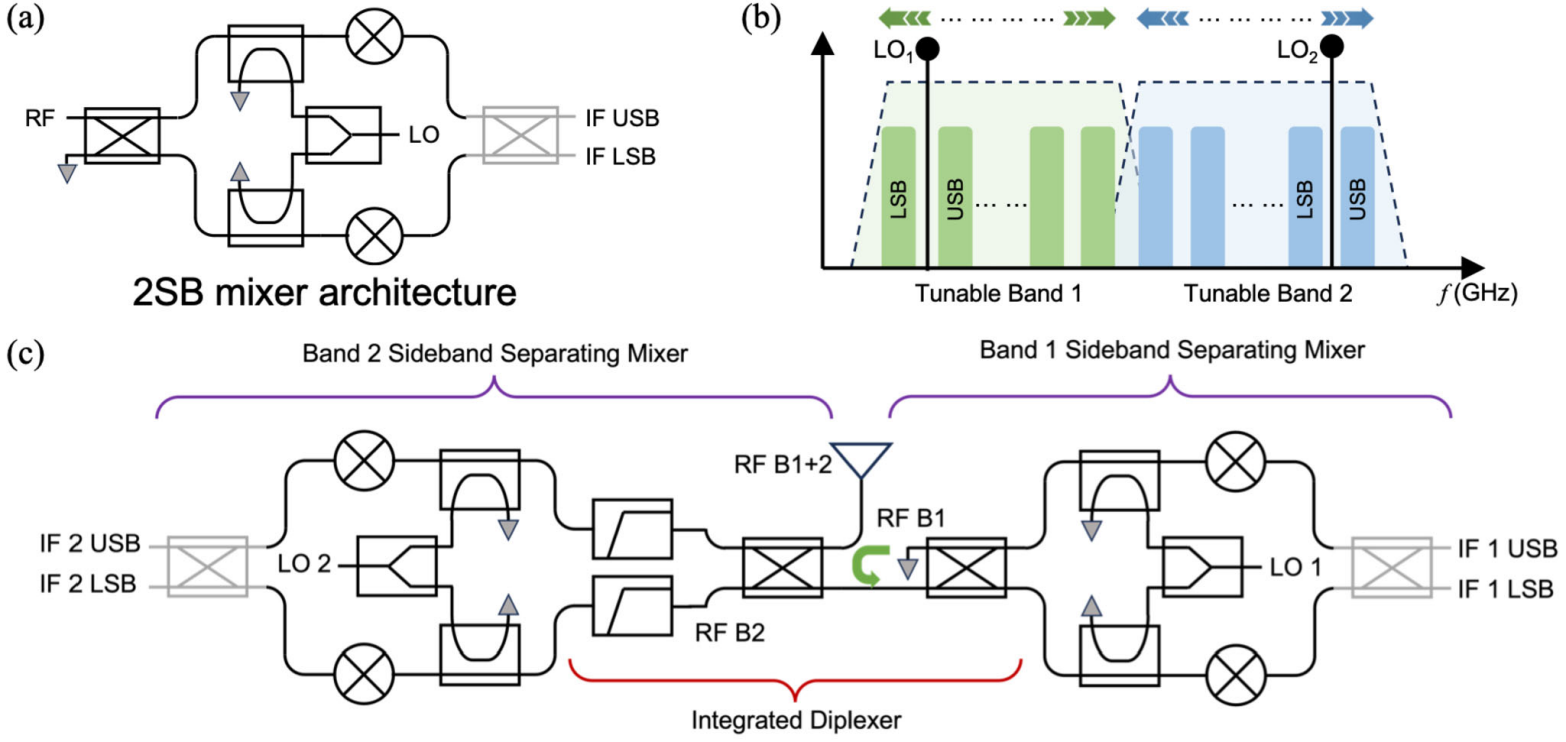
(a) Recap of the 2SB mixer architecture for reference. (b) Illustration of how the proposed SOMBR scheme enables LO tuning within a dual-band architecture to recover the full RF bandwidth. (c) Schematic of the dual-band SOMBR, highlighting the required components. The architecture closely resembles two back-to-back 2SB mixers as shown in (a), with the addition of a pair of high-pass filters

The proposed dual-band SOMBR architecture is illustrated in Fig. 16. For illustration, we consider two arbitrary bands, denoted Band 1 and Band 2. One immediately notices that the right-hand portion of Fig. 16(c) closely resembles a conventional 2SB receiver, as shown in Fig. 7. The left-hand portion similarly follows a 2SB architecture, with the addition of a pair of HPFs to define the frequency band. This configuration highlights the conceptual simplicity of the approach, whereby two standard 2SB receivers are combined through a minimal filtering stage to realise dual-band operation.

A key advantage of this scheme is the retention of full frequency agility. Since each band is processed by an independent 2SB receiver, the LO frequencies can be tuned arbitrarily within their respective RF ranges, enabling continuous coverage of the accessible spectrum, as illustrated in Fig. 16(b). This represents a significant improvement over fixed-band diplexing schemes. Furthermore, compared to the existing implementation shown in Fig. 15, the proposed architecture requires only a single set of quadrature hybrids and a minimal number of filtering elements, rather than multiple cascaded diplexer stages. When combined with planar circuit implementations of the hybrid components, this results in a substantially more compact and scalable system.

The central innovation of this approach lies in reusing the RF quadrature hybrids inherent to the 2SB architecture, together with only two additional filtering elements, to realise an integrated diplexer. In practice, these HPFs can be implemented directly within the existing waveguide sections used to feed the quadrature hybrids, by appropriately selecting their dimensions to exploit the waveguide cutoff frequency. As a result, no fundamentally new components are required beyond those already present in a conventional 2SB receiver. Instead, the SOMBR functionality is achieved through a reconfiguration of existing elements, leading to a highly efficient and compact design.

Since the design and operation of 2SB SIS mixers have been extensively discussed in Sect. 3.4, the following sections focus on the design and electromagnetic simulation of the integrated diplexer stage, highlighted in red curly bracket in Fig. 16(c).

### ALMA band 6+7 integrated diplexer

**Fig. 17:**
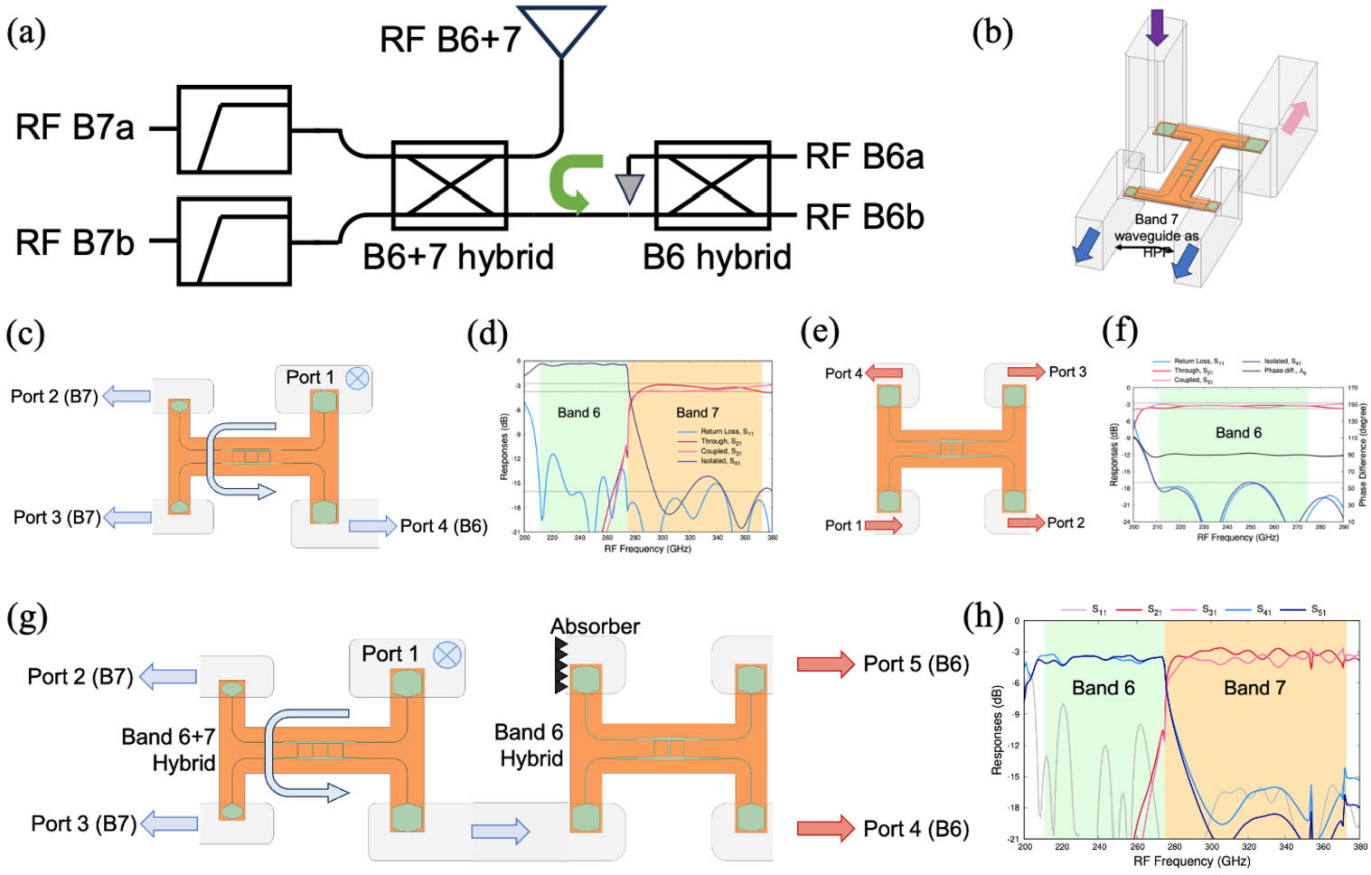
(a) Schematic layout of the integrated Band 6+7 hybrid diplexer. (b) Three-dimensional illustration showing the mounting of the planar superconducting hybrid chip within a waveguide block. (c) Top-down view of the Band 6+7 hybrid, employing narrower rectangular waveguide sections at the output ports to realise high-pass filtering. (d) Simulated performance of the Band 6+7 hybrid with integrated straight-waveguide HPFs. (e) Top-down view of the Band 6 hybrid. (f) Simulated performance of the Band 6 hybrid. (g) Top-down view of the complete integrated diplexer, comprising the Band 6+7 hybrid, waveguide HPF sections, and the Band 6 hybrid. (h) Simulated performance of the integrated diplexer shown in (g)

Figure 17 illustrates the design and simulated performance of a dual-band integrated diplexer, using the ALMA-defined Band 6 and Band 7 frequency ranges as a representative example. The electromagnetic performance of the proposed structure is evaluated using Ansys High Frequency Structure Simulator (HFSS), providing a realistic assessment of its feasibility.

Figure 17(a) presents the overall architecture of the Band,6+7 integrated diplexer. The system comprises a dual-band antenna, a broadband B6+7 quadrature hybrid, a Band 6-specific hybrid stage,^9^ and a pair of high-pass filters (HPFs). Figure 17(b) and (c) show the construction of the dual-band quadrature hybrid, realised by cascading three branch-line couplers to achieve broadband performance. The hybrid chip, fabricated using standard Nb transmission-line technology, is mounted at the split-plane of a waveguide block comprising four channels. Broadband probe antennas are employed to interface between the planar circuitry and the waveguide structure.

As expected, the RF hybrid layout closely resembles that described in Sect. 3.4.2, reflecting the reuse of standard 2SB receiver components within the SOMBR architecture. The key distinction lies in the design of the output waveguide ports (ports 2 and 3, corresponding to the through and coupled ports), which are dimensioned to introduce a cutoff frequency at the boundary between Band 6 and Band 7. As a result, signals within Band 7 propagate through these ports with approximately equal power splitting ($\sim -3\text{ dB}$), while lower-frequency Band 6 signals are reflected. These reflected components recombine at the isolated port (port 4), thereby achieving frequency-selective separation of the broadband input signal.

The simulated performance of the dual-band hybrid combined with the HPF structure is shown in Fig. 17(d). The results indicate that Band 7 signals are efficiently transmitted through ports 2 and 3, with power splitting close to $-3\text{ dB}$ and amplitude imbalance within approximately 1 dB across the band. The recombined Band 6 signal is directed to port 4 with minimal loss. The return loss remains better than $-12\text{ dB}$ across the full Band,6+7 frequency range.

Figure 17(e) and (f) show the layout and performance of the Band 6 hybrid stage, in which all four waveguide channels share identical dimensions. Owing to its narrower operational bandwidth, this stage achieves improved performance, with amplitude imbalance maintained within 1 dB of the ideal $-3\text{ dB}$ split and return loss better than $-17\text{ dB}$ across Band 6.

Finally, Fig. 17(g) presents the complete integrated diplexer assembly, combining the broadband Band 6+7 hybrid, the HPF sections, and the Band 6 hybrid stage. The expected signal flow is as follows: Band 6 signals enter through port 1, propagate through the broadband hybrid, are reflected by the HPF waveguide sections, recombine at the isolated port, and are subsequently split by the Band 6 hybrid into ports 5 and 6. In contrast, Band 7 signals propagate directly through the broadband hybrid and are equally divided between ports 2 and 3. The isolated port of the Band 6 hybrid is ideally terminated with an absorber, although this termination is not included in the present simulation.

The simulated performance of the complete system is shown in Fig. 17(h). While the amplitude imbalance is slightly degraded compared to the individual components, and the return loss within Band 6 exhibits some localised degradation, the overall performance remains promising. This example demonstrates the practical feasibility of the integrated diplexer concept, while also indicating that further optimisation through more detailed electromagnetic design and analysis is likely to yield improved performance.

### Tri-band and beyond SOMBR scheme

In principle, the dual-band SOMBR concept described above can be extended by cascading multiple stages to realise a pan-chromatic, multi-band receiver capable of simultaneous observation across several frequency bands. In such an architecture, each stage incorporates filters with distinct cutoff frequencies, progressively partitioning the incoming broadband RF signal into multiple sub-bands.

**Fig. 18:**
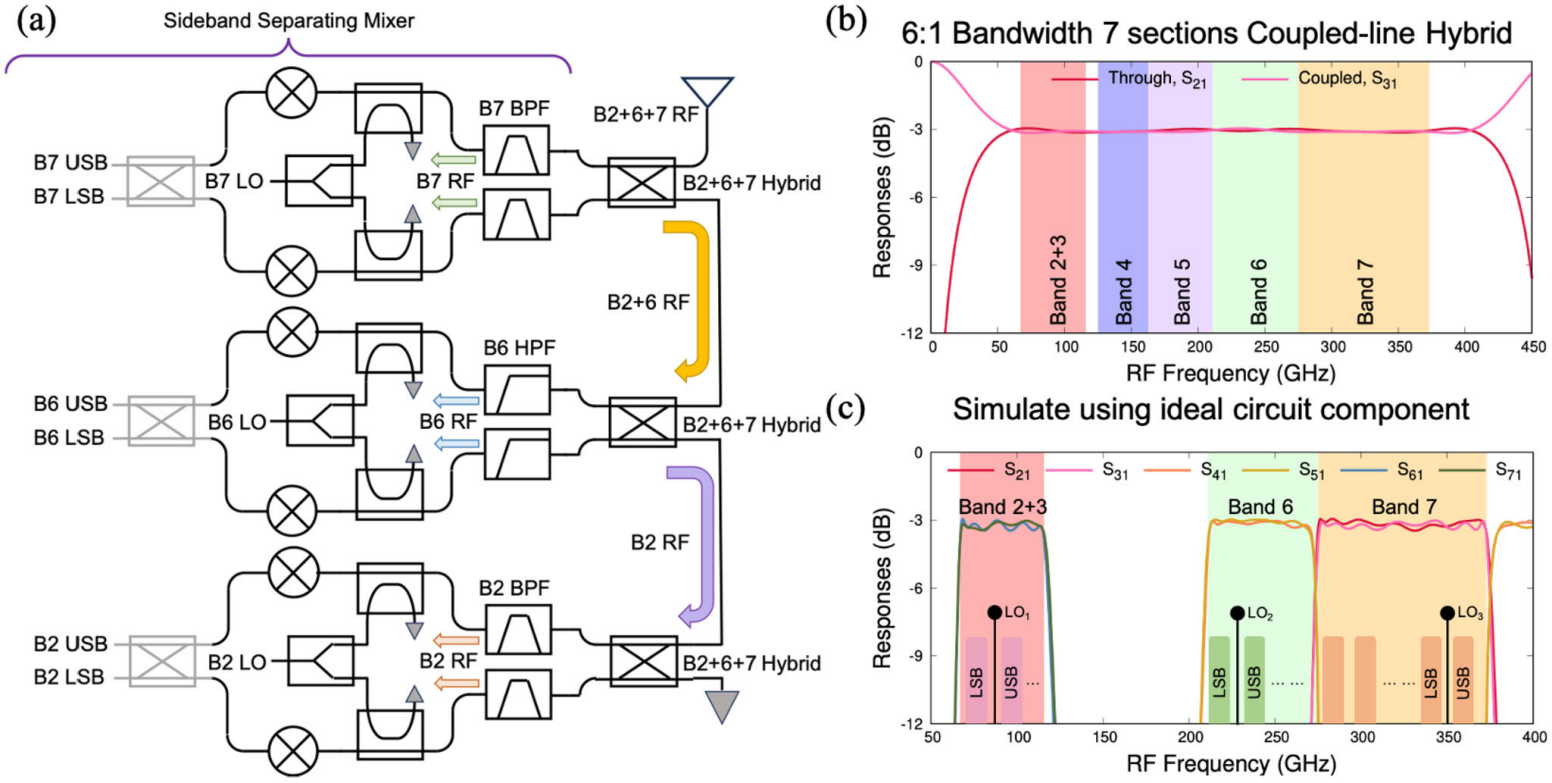
(a) Schematic illustrating the architecture of a tri-band SOMBR covering ALMA Bands 2, 6, and 7. (b) Simulated performance of a seven-section coupled-line hybrid operating across Bands 2–7. (c) Simulated performance of the tri-band SOMBR using idealised components

Figure 18 illustrates a tri-band SOMBR configuration, using ALMA Bands 2, 6, and 7 as an example, corresponding to the first three rotational transitions of carbon monoxide. This implementation requires operation over a broad frequency range of approximately 67–373 GHz, corresponding to a fractional bandwidth of nearly 6:1. Consequently, both the input antenna and the first-stage quadrature hybrid must support ultra-broadband operation. Subsequent hybrid stages may be designed with narrower bandwidths (e.g., Band 2+6 for the second stage and Band 2 for the third stage); however, for simplicity, a broadband hybrid is assumed for all stages in this illustrative example.

In this architecture, the incoming astronomical signal is first received by an ultra-broadband antenna and fed into the initial quadrature hybrid. At this stage, the highest-frequency band (Band 7) is transmitted to the corresponding 2SB receiver. The remaining lower-frequency components (Band 2+6) are reflected, recombine within the hybrid, and are subsequently directed to the second stage. There, Band 6 is separated and routed to a second 2SB receiver, while the remaining Band 2 signal is reflected, recombined, and directed to a third 2SB receiver. In principle, the filtering at each stage may be implemented using either high-pass filters or low-pass filters; in this example, band-pass filters are introduced at selected stages to improve band definition. In practice, the choice of filter topology has limited impact on in-band performance, provided that adequate selectivity is achieved.

To demonstrate the feasibility of this concept, we perform circuit-level simulations using ideal components in the Keysight Advanced Design System (ADS). To accommodate the required ultra-broadband operation, conventional branch-line couplers are replaced with multi-section coupled-line hybrids. Figure 18(b) shows the simulated performance of a seven-section coupled-line hybrid, which demonstrate an ideal $-3\text{ dB}$ power splitting across the full 67–373 GHz range. It should be noted that these results are based on idealised circuit elements and do not account for parasitic effects or fabrication constraints, and therefore represent an upper bound on achievable performance.

**Fig. 19:**
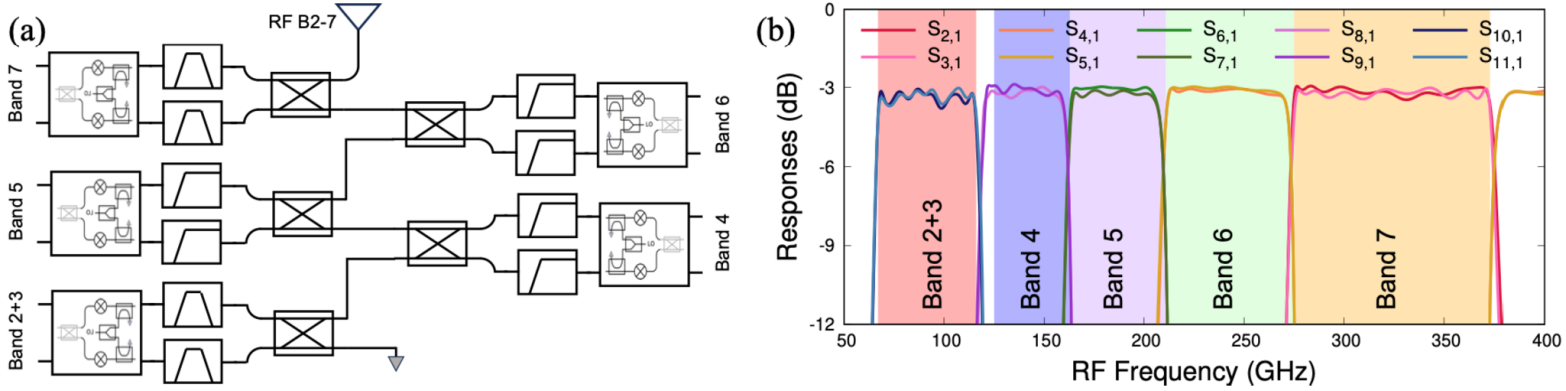
(a) Schematic illustrating the architecture of a five-band SOMBR covering the RF range from ALMA Bands 2–7. (b) Simulated performance of the five-band SOMBR using idealised components

The simulated performance of the complete tri-band SOMBR system is shown in Fig. 18(c), demonstrating effective separation of the RF spectrum into the three target bands. While a gap between Bands 2 and 6 is evident in this configuration, this can be readily addressed by introducing additional intermediate stages, as illustrated in Fig. 19. In this extended architecture, additional 2SB receivers are inserted between Bands 2 and 6, enabling continuous and simultaneous coverage across multiple ALMA bands.

**Fig. 20:**
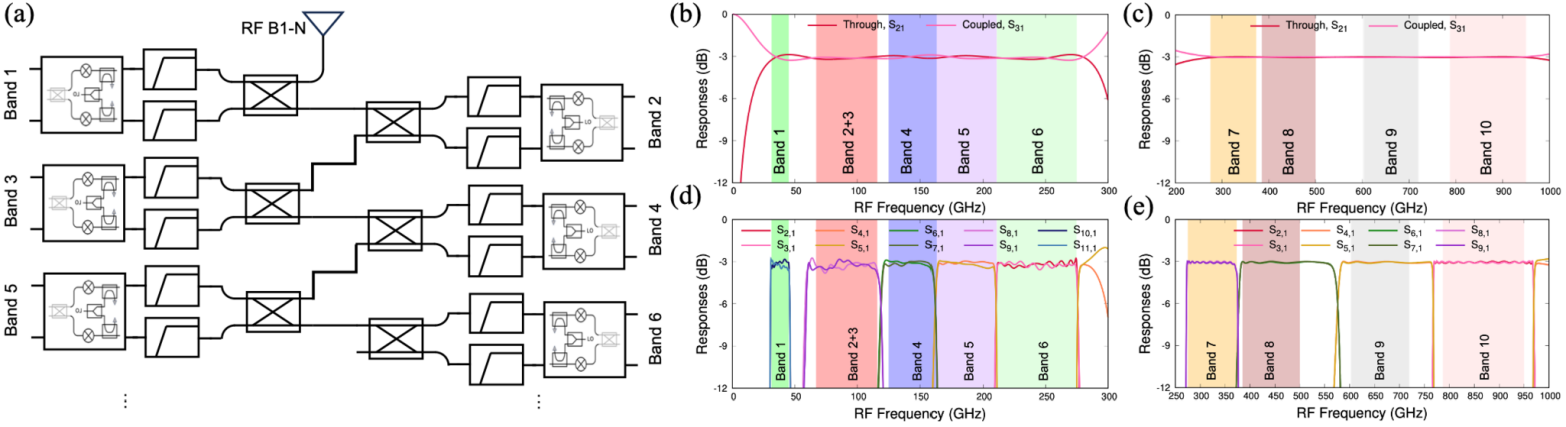
(a) Schematic illustrating the architecture of a multi-band SOMBR. Simulated performance of the coupled-line hybrids for (b) Bands 2–6 and (c) Bands 7–10. (d) and (e) Simulated performance of the lower- and upper-band SOMBR implementations, respectively

This concept can be generalised further to achieve near-continuous coverage across the full ALMA frequency range (30–950 GHz), as illustrated in Fig. 20. For practical implementation, the system may be partitioned into multiple sub-receivers (e.g., Bands 1–6 and Bands 7–10 SOMBR), each covering a manageable bandwidth range.

It is important to emphasise that such ultra-broadband architectures present significant technical challenges. Components operating across wide fractional bandwidths must maintain high performance in terms of insertion loss, amplitude balance, and phase accuracy. Nevertheless, wideband receiver concepts with bandwidth ratios approaching or exceeding an octave have been demonstrated at lower frequencies (Kooi et al. 2023), including the deployment of ultra-wideband feed structures that could be coupled to the proposed ultra-wideband planar hybrid through appropriately designed broadband on-chip antennas. These demonstrations indicate the feasibility of such approaches in principle, although extending them to the mm/sub-mm regime will require further advances in materials, fabrication techniques, and electromagnetic design. If realised, ultra-broadband SOMBR architectures could enable continuous, high-resolution spectroscopic observations across the mm/sub-mm spectrum, potentially offering a compact and high-sensitivity alternative to ultra-broadband bolometric spectrometers, with the added advantage of intrinsic high spectral resolution.

### Short summary

In this section, we have introduced a novel concept for simultaneous observing multi-band receivers (SOMBRs), enabling concurrent 2SB operation across a wide RF bandwidth. This approach offers capabilities analogous to on-chip filterbank spectrometers, but with substantially higher spectral resolution and, crucially retains phase information. This makes SOMBR architectures uniquely suited for interferometric applications, such as those required for EHT observations.

It is important however, to recognise that SOMBRs are not universally superior to alternative ultra-broadband spectrometer technologies. Bolometric spectrometers, including on-chip filterbank and Fourier-transform variants, can in principle achieve lower noise performance, as they are not constrained by the quantum noise limit inherent to heterodyne detection. Consequently, the choice of spectrometer architecture should be guided by the specific scientific application, balancing requirements such as spectral resolution, sensitivity, bandwidth, and compatibility with interferometric techniques.

Hybrid approaches are also emerging, such as filterbank architectures employing SIS mixers in place of bolometric detectors (Groppi et al. 2019), which may combine ultra-high spectral resolution with phase-sensitive detection capabilities. Furthermore, the SOMBR concept is not limited to SIS technology and could be extended to other heterodyne detectors, including Schottky diodes and hot-electron bolometers (HEBs), broadening its applicability across different frequency regimes.

Looking ahead, the SOMBR architecture may be further refined through the integration of all 2SB receiver components onto superconducting planar circuits, significantly reducing system footprint and enabling scalable implementations. This, in turn, opens the possibility of extending SOMBR concepts to large-format arrays, providing simultaneous multi-band, high-resolution spectroscopic capability over wide fields of view. Ultimately, the concepts presented here suggest a pathway towards increasingly compact, broadband, and functionally rich heterodyne receiver systems. Further innovation e.g., potentially combining ideas from both heterodyne and bolometric technologies, will be required to realise the full potential of ultra-broadband, high-resolution instruments for future mm/sub-mm astronomy.

## Conclusion

In this work, we have examined the prospects and challenges associated with the development of next-generation astronomical mm/sub-mm and terahertz heterodyne receivers. Building on the established success of SIS-based instrumentation, we have outlined a series of technological pathways aimed at substantially enhancing receiver capability in terms of bandwidth, functionality, and scalability.

A central theme of this manuscript is the transition from single-pixel, narrowly optimised receiver systems towards highly integrated, multifunctional architectures. In particular, we have explored approaches to achieving ultra-broad RF and IF bandwidth SIS mixers, compact sideband-separating (2SB) receiver designs through planar superconducting circuit integration, and scalable focal plane array (FPA) architectures capable of supporting hundreds to thousands of pixels. These developments are motivated by the increasing demand for high-throughput, wide-field, and high-spectral-resolution observations across the mm/sub-mm and THz regimes.

We have further introduced the concept of simultaneous observing multi-band receivers (SOMBRs), demonstrating how conventional 2SB architectures can be reconfigured to enable concurrent multi-band observations with minimal additional hardware. This approach offers a compelling pathway towards continuous, high-resolution spectroscopic coverage across broad frequency ranges, while retaining compatibility with interferometric techniques. Together with advances in planar circuit integration and vertical array architectures, such concepts point towards a new generation of compact, high-performance receiver systems.

Despite these promising developments, significant technical challenges remain. Achieving simultaneous ultra-broad RF and IF bandwidth requires careful management of competing design constraints, particularly those arising from junction capacitance, impedance matching, and transmission line parasitics. Scaling receiver architectures to large-format arrays introduces additional complexity in local oscillator distribution, interconnect density, thermal management, and system integration. Furthermore, the realisation of compact, fully integrated 2SB and multi-band architectures demands continued advances in fabrication precision, materials, and electromagnetic design. At higher frequencies, particularly beyond the superconducting gap of niobium, further challenges arise due to increased losses and the need for alternative materials such as NbTiN or NbN. Addressing these limitations will be essential for extending heterodyne receiver performance into the supra-THz regime.

Looking forward, continued progress in this field will rely on coordinated advances across multiple domains, including superconducting device physics, micro-fabrication, circuit design, and system engineering. Emerging technologies—such as ultra-thin membrane substrates, high critical current density junctions, and innovative planar coupling structures—offer promising avenues for overcoming current limitations. At the same time, hybrid approaches that combine elements of heterodyne and bolometric detection may further expand the accessible parameter space for future instrumentation.

The scientific drivers for these developments are compelling. Future facilities, including ALMA upgrades, AtLAST, LST, and next-generation space missions, will require receiver technologies that deliver orders-of-magnitude improvements in mapping speed, bandwidth, and sensitivity. In parallel, interferometric programmes such as the EHT and its successors will benefit from multi-band, phase-sensitive observing capabilities enabled by advanced heterodyne systems.

In conclusion, while the challenges are substantial, the opportunities for innovation in mm/sub-mm and THz heterodyne receiver technology are equally significant. The concepts and approaches presented in this manuscript illustrate that a pathway exists towards highly integrated, ultra-broadband, and large-format receiver systems. Realising this vision will require sustained effort and collaboration across the community, but holds the promise of enabling transformative advances in our understanding of the Universe.

## Data Availability

No datasets were generated or analysed during the current study.

## Footnotes

1 Following recent developments, Bands 2 and 3 have been combined into a single receiver cartridge; consequently, ALMA is currently equipped with nine physical receiver units.

2 The lower frequency limit is defined by a return loss of –6, dB, ensuring stable mixer operation, while the upper limit is set by the frequency at which the noise temperature exceeds the ALMA-WSU specification.

3 In this design, part of the RF choke circuitry is implemented using coplanar waveguide (CPW) transmission lines rather than microstrip, and the ground plane beneath the bonding pads is removed to minimise parasitic microstrip capacitance.

4 Note that in this case, unlike the mixer design shown in Fig. 1 (a), we replace part of the RF-choke circuits with coplanar waveguide (CPW) transmission line instead of microstrip, as well as removing the ground plane of the bonding pads to minimise the microstrip structures that may impact the IF bandwidth performance.

5 The device shown in Fig. 4(d) is fabricated on an 80 μm thick quartz substrate, rather than a thin SoI membrane.

6 Application of travelling wave parametric amplifier in astronomical receiver could potentially revolutionise the architecture of heterodyne receiver, among many other applications, but discussion of this topic is beyond the scope of this manuscript.

7 In the following, these RF components excluding the IF quadrature hybrid are collectively referred to as the “hybrid block”.

8 An equivalent configuration can be realised by injecting the RF in phase and introducing a 90^∘^ phase shift in the LO path.

9 This stage could alternatively employ a broadband Band,6+7 hybrid; however, narrower-band components typically offer improved performance. In this example, the lower-frequency band is selected for backward reflection-operation, although the approach is equally applicable to select Band 7 for backward reflection-operation, in which case the HPFs would be replaced by low-pass filters (LPFs).
